# Glutathione‐Sensitive Photosensitizer–Drug Conjugates Target the Mitochondria to Overcome Multi‐Drug Resistance in Cancer

**DOI:** 10.1002/advs.202307765

**Published:** 2024-06-19

**Authors:** Weiguo Song, Hekai Yang, Ying Wang, Shuzhen Chen, Wenda Zhong, Qian Wang, Wenshuo Ding, Guangzhao Xu, Chen Meng, Ying Liang, Zhe‐Sheng Chen, Shuhua Cao, Liuya Wei, Fahui Li

**Affiliations:** ^1^ Department of Medicinal Chemistry School of Pharmacy Shandong University Jinan 250012 China; ^2^ School of Pharmacy Weifang Medical University Weifang 261053 China; ^3^ Weifang Synovtech New Material Technology CO., LTD. Weifang 262700 China; ^4^ Harway Pharma Co., Ltd. Dongying 254753 China; ^5^ Department of General Practice The First Affiliated Hospital of Shandong First Medical University Jinan 250013 China; ^6^ Department of Pharmaceutical Sciences College of Pharmacy and Health Sciences St. John's University Queens NY 11439 USA; ^7^ College of Chemistry Chemical and Environmental Engineering Weifang University Weifang 261061 China

**Keywords:** cancer multi‐drug resistance, GSH sensitive, mitochondria ‐ targeting, photodynamic therapy, the combination of photodynamic therapy and chemotherapy

## Abstract

Multi‐drug resistance (MDR) is a major cause of cancer therapy failure. Photodynamic therapy (PDT) is a promising modality that can circumvent MDR and synergize with chemotherapies, based on the generation of reactive oxygen species (ROS) by photosensitizers. However, overproduction of glutathione (GSH) by cancer cells scavenges ROS and restricts the efficacy of PDT. Additionally, side effects on normal tissues are unavoidable after PDT treatment. Here, to develop organic systems that deliver effective anticancer PDT and chemotherapy simultaneously with very little side effects, three GSH‐sensitive photosensitizer‐drug conjugates (CyR‐SS‐L) are designed and synthesized. CyR‐SS‐L localized in the mitochondria then is cleaved into CyR‐SG and SG‐L parts by reacting with and consuming high levels of intracellular GSH. Notably, CyR‐SG generates high levels of ROS in tumor cells instead of normal cells and be exploited for PDT and the SG‐L part is used for chemotherapy. CyR‐SS‐L inhibits better MDR cancer tumor inhibitory activity than indocyanine green, a photosensitizer (PS) used for PDT in clinical applications. The results appear to be the first to show that CyR‐SS‐L may be used as an alternative PDT agent to be more effective against MDR cancers without obvious damaging normal cells by the combination of PDT, GSH depletion, and chemotherapy.

## Graphical abstract

A glutathione‐sensitive photosensitizer‐drug is firsted designed to simultaneously realize tumor photodynamic therapy and chemotherapy. The disulfide linkage in predrug is broken by glutathione reductase, releasing active photosensitizers and Lonidamine. Due to the synergistic effect, the expression of ATP‐binding cassette subfamily B member 1 (ABCB1) is reduced and the drug resistance pathway is broken, resulting to the efficient cancer treatment.

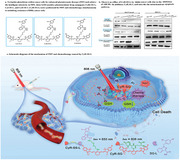

## 1 Introduction

Multi‐drug resistance (MDR) to chemotherapeutic agents and molecularly targeted drugs are responsible for most cancer recurrence, which leads to approximately 80–90% of cancer‐related deaths.^[^ ^1^, ^2^, ^3^ ^]^ The most common mechanism responsible for MDR development involves the overexpression of the adenosine triphosphate (ATP) binding cassette (ABC) transporters that extrude chemotherapy drugs from cancer cells, including P‐glycoprotein P‐gp, ATP‐binding cassette subfamily B member 1 (ABCB1, P‐gp, or MDR1), breast cancer resistant protein (BRCP or ABCG2), and multidrug resistance‐related proteins 1‐6 (MRP1‐6 or ABCC1‐6).^[^ ^4^, ^5^ ^]^ The most well‐known drug efflux transporter linked to MDR, ABCB1 is associated with a variety of cancers that are resistant to many currently used anti‐cancer drugs, such as paclitaxel, vinblastine, and daunorubicin,^[^ ^6^ ^]^ and MDR‐mediated by ABCB1 is one of the main barriers for the success of cancer treatment. Inhibition of ABCB1 or down‐regulation of its expression has proven to be one of the first approaches to surmounting MDR.^[^ ^7^ ^]^

Photodynamic therapy (PDT) is a novel cancer treatment that has high selectivity, minimal invasiveness, and limited side effects.^[^ ^8^, ^9^, ^10^, ^11^ ^]^ PDT consists of three key elements: photosensitizer (PS), a light source, and oxygen, as the source of ROS.^[^ ^12^, ^13^, ^14^ ^]^ When the PSs accumulate in the tumor site, irradiation at a specific laser wavelength produces cytotoxic ROS,^[^ ^15^, ^16^ ^]^ which then kill cancer cells by apoptosis or necrosis and inhibits tumor growth.^[^ ^17^, ^18^ ^]^ Cyanine dyes such as indocyanine green (ICG) have been employed as effective PS for PDT due to their intrinsic mitochondrial targeting ability and strong ROS‐producing capacity.^[^ ^19^ ^]^ At present, the selectivity of PDT is achieved by manually controlling the irradiation site. However, in clinical practice, there is always a portion of light irradiation on normal tissues, resulting in treatment‐related toxicity and side effects. Therefore, it is very urgent to achieve intelligent selectivity in PDT. ICG can be used as PS to generate ROS and damage cancer cells by light illumination,^[^ ^20^, ^21^ ^]^ ICG has some limitations such as short half‐life, poor photo‐stability, and lack of specific targeting,^[^ ^21^ ^]^ restricting its application in cancer treatment. Moreover, the high concentration of GSH in cancer cells can eliminate the ROS produced, consequently restricting the therapeutic efficacy of PDT.^[^ ^22^ ^]^

In recent years, PDT is used in synergy with other therapeutic methods (such as chemotherapy or immunotherapy) to inhibit tumor growth.^[^ ^23^, ^24^ ^]^ One promising combination includes the chemotherapy drug, lonidamine (LNDM), which may enhance the effect of PSs. Clinically, LNDM targets a variety of tumor cells with little toxicity and few side effects to normal cells and is usually combined with other drugs or treatments to enhance the therapeutic effect.^[^ ^25^ ^]^ A mitochondrial‐targeted LNDM created by linking LNDM to triphenylphosphine was found to induce ROS production.^[^ ^26^, ^27^ ^]^ Furthermore, LNDM prevents glycolysis by inhibiting hexokinase II (HK‐II), thereby reducing NADPH and GSH levels.^[^ ^28^, ^29^ ^]^ Moreover, LNDM can inhibit aerobic respiration in tumor cells and mitigate the effects of tumor hypoxia on PDT efficiency.^[^ ^30^, ^31^ ^]^ The combined use of LNDM and 5‐aminolevulinic acid (ALA) has also been shown to enhance the therapeutic effect of PSs.^[^ ^32^ ^]^

The distinct mechanisms of chemotherapy and PDT can overcome the drawbacks of single PDT treatment, such as limited tissue penetration of light and low ROS production.^[^ ^33^, ^34^, ^35^ ^]^ However, simultaneous delivery of PSs and small molecule drugs to tumor sites is difficult, because they have markedly different circulation half‐lives, which makes it difficult for both molecules to accumulate in the tumor site at the same time.^[^ ^36^ ^]^ Current existing nanoplatforms used for combined therapies simply co‐load PSs and anticancer drugs to nanocarriers. The preparation process of such a nano‐drug delivery system is complicated, and carrier materials have potential toxicity if they cannot be degraded over time. Moreover, nano‐drug delivery systems often have problems such as poor drug loading capacity and drug leakage during delivery, causing adverse reactions.^[^ ^37^, ^38^, ^39^, ^40^ ^]^ Therefore, new organic systems must be developed that effectively deliver PDT and chemotherapy simultaneously.

**Scheme 1:**
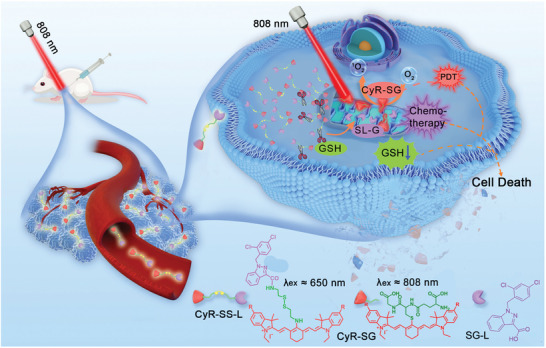
Schematic representation of photodynamic therapy and chemotherapy mechanism induced by CyR‐SS‐L cleavage by GSH.

Here, we designed and synthesized three GSH‐sensitive PSs‐drug conjugates CyH‐SS‐L, CyBr‐SS‐L, and CyI‐SS‐L for PDT and chemotherapy. As shown in **Scheme** **1**, the conjugate consists of indole cyanine dye and LNDM linked by cystamine dihydrochloride. The conjugate targets cancer cells through the LNDM group and is localized to mitochondria by indole cyanine dye after entering tumor cells. Under the high levels of GSH in mitochondria, the conjugate is cleaved into two parts, cyanine dye (CyR‐SG) and SG‐L (LNDM group). Our observations support that this strategy has clear advantages: 1) The PSs moiety, CyR‐SG, is used for PDT and the introduction of heavy atoms in CyR‐SS‐L markedly elevated the yield of ROS, which was only produced at the tumor site and not in normal tissues; 2) The intracellular GSH was significantly decreased by CyR‐SS‐L leading to the high efficacy of PDT; 3) The SG‐L moiety acted as chemotherapy, which enhanced the effect of PDT. Hence, CyR‐SS‐L showed excellent anti‐cancer effects in vitro and in vivo by exerting synergistic effects of PDT, GSH depletion, and chemotherapy.

## 2 Results

### 2.1 Synthesis and Characterization of CyR‐SS‐L

The compounds were synthesized by a multistep reaction (Scheme S1, Supporting Information). The heavy atoms were introduced in the quaternary ammonium structure of indole cyanine dye, which could enhance the spin‐orbit coupling and eventually boost ROS production. And the antineoplastic drug LNDM was linked by disulfide bonds in the meso‐position of cyanine dye. All intermediates and CyR‐SS‐L were characterized by nuclear magnetic resonance (NMR) and mass spectrometry (Figures S1–S9, Supporting Information).

### 2.2 GSH Successfully Binds to the Disulfide Bond of CyR‐SS‐L Photosensitizers, Leading to Its Reduction

**Figure 1:**
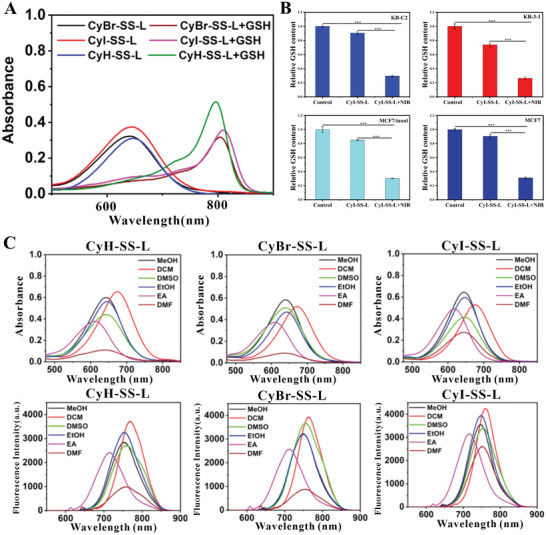
The extracellular and intracellular GSH content was significantly reduced by CyBr‐SS‐L, CyI‐SS‐L, and CyH‐SS‐L PSs. A) UV absorption spectra of PSs before and after GSH treatment in DMSO solution. B) The content of intracellular GSH in KB‐3‐1, KB‐C2, MCF7, and MCF7/taxol cells treated with CyI‐SS‐L. C) UV–vis absorption and fluorescence spectra of CyH‐SS‐L, CyBr‐SS‐L, or CyI‐SS‐L in MeOH, DCM, DMSO, EtOH, EA, and DMF solvent (***p < 0.001).

The disulfide bonds within CyR‐SS‐L PSs facilitate binding with GSH and conjugation with chemotherapy agents via disulfide linkages. Upon entry into cancer cells, CyR‐SS‐L PSs form bonds with GSH through disulfide linkages, thereby depleting the intracellular pool of GSH. To validate this reaction, we simulated a high GSH extracellular milieu in solution. In the presence of GSH, the UV absorption spectrum of CyR‐SS‐L undergoes a pronounced red shift, transitioning from an initial absorption peak near 660 nm to approximately 800 nm (**Figure** **1**A). This spectral shift arises from the reaction of CyR‐SS‐L with excess GSH, yielding CyR‐SG and SG‐L, as depicted in Figure S10 (Supporting Information), and confirmed via mass spectrometric analysis of CyR‐SS‐L in GSH solution (Figures S11–S13, Supporting Information). The sequential process unfolds as follows: within the high GSH environment of the cellular milieu, the conjugate undergoes cleavage, yielding CyR‐SH and the SG‐L. Subsequently, CyR‐SH further reacts with GSH to generate CyR‐SG, manifesting a maximum absorption wavelength shift from approximately 650 nm (CyR‐SS‐L) to around 800 nm. This phenomenon can be explained by molecular structure perspective, when CyR‐SS‐L is converted to CyR‐SG (Figure S10, Supporting Information), the atom connected to the cyanine dye at the midpoint changed from N to S (from electron donating groups to electron withdrawing groups), thereby affecting intramolecular charge transfer and causing the absorption peak to shift towards longer wavelengths, from 650 to 808 nm.

The maximum absorption peak of CyR‐SG is around 800 nm. Due to the low content of GSH in normal cells, the maximum absorption peak of CyR‐SS‐L remained around 650 nm. Therefore, when cells were irradiated with 808 nm, a large amount of ROS was produced in cancer cells compared with normal cells. This change in absorption wavelength is of great significance for achieving intelligent selectivity of PDT. Subsequently, we determined the amount of GSH bound to CyI‐SS‐L based on the level of intracellular GSH content in KB‐3‐1, KB‐C2, MCF7 and MCF7/taxol cells. As shown in Figure 1B, CyI‐SS‐L consumed a large amount of GSH under light conditions. Moreover, the UV visible and fluorescence spectra of CyH‐SS‐L, CyBr‐SS‐L, and CyI‐SS‐L in MeOH, DCM, DMSO, EtOH, EA, and DMF were studied. As shown in Figure 1C, the UV absorption peaks of CyR‐SS‐L PSs are located in the near‐infrared region, and the light in the near‐infrared region has relatively deep tissue penetration, which is significant for further clinical use.

### 2.3 CyR‐SS‐L PSs Have a Significant Inhibitory Effect on Parental Cancer Cells and MDR Cancer Cells Overexpressing ABCB1

**Figure 2:**
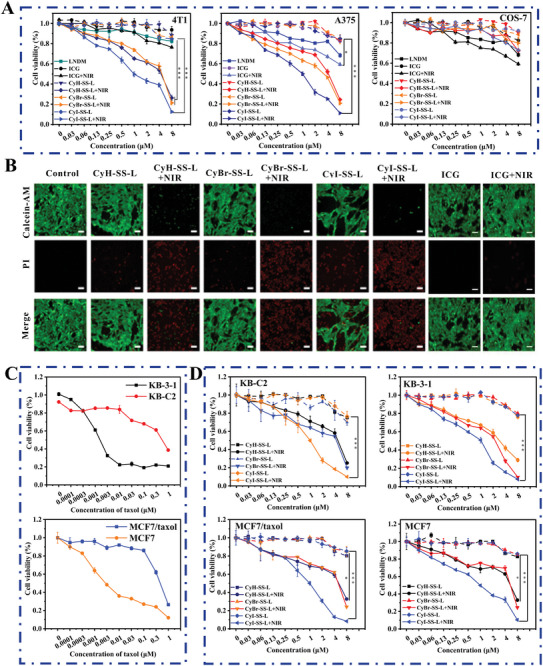
CyR‐SS‐L photosensitizer has a significant inhibitory effect on MDR cancer cells overexpressing ABCB1, as well as the parental cancer cells and 4T1 and A375 cells. A) The anti‐proliferation effect of CyBr‐SS‐L, CyI‐SS‐L, and CyH‐SS‐L on 4T1 and A375 cells. Cells were stimulated with CyBr‐SS‐L, CyI‐SS‐L, or CyH‐SS‐L combined with or without light irradiation (808 nm, 200 mW cm^−2^, 1 min). B) Live‐dead imaging of 4T1 cells treated with CyBr‐SS‐L, CyI‐SS‐L, or CyH‐SS‐L. (Calcein AM: *λ*_ex_: 488 nm and *λ*_em_: 500–580 nm; PI: *λ*_ex_: 561 nm and *λ*_em_: 600–700 nm). Scale bars: 150 µm. C) The anticancer efficacy of paclitaxel on MCF7, MCF7/taxol, KB‐3‐1, KB‐C2 cells. D) The anti‐proliferation effect of CyBr‐SS‐L, CyI‐SS‐L, or CyH‐SS‐L on MCF7, MCF7/taxol, KB‐3‐1, and KB‐C2 cells. Cells were treated with CyBr‐SS‐L, CyI‐SS‐L or CyH‐SS‐L combined with or without light irradiation (808 nm, 200 mW cm^−2^, 1 min). (*p < 0.05, **p < 0.05, ***p < 0.001). Scale bars: 200 µm.

The anti‐proliferative effects of CyR‐SS‐L (CyH‐SS‐L, CyBr‐SS‐L, and CyI‐SS‐L) PSs on 4T1, A375, and COS‐7 cells were evaluated under both dark and light conditions. As depicted in **Figure** **2**A, in the absence of light, cell viability of CyH‐SS‐L, CyBr‐SS‐L, and CyI‐SS‐L on cancer cells remained above 85% when the compound concentration was below 8 × 10^−6^ m, which indicated relatively low dark toxicity of CyR‐SS‐L. Upon irradiation at 808 nm, the IC_50_ values of CyI‐SS‐L for 4T1 and A375 cells were 0.84 × 10^−6^ m and 1.00 × 10^−6^ m, respectively. CyH‐SS‐L and CyBr‐SS‐L also exhibited inhibitory effects on the proliferation of 4T1 and A375 cells. It can be seen that the anti‐proliferation of CyR‐SS‐L combined with irradiation at 808 nm was highly potent than LNDM. Intriguingly, even under 808 nm irradiation, the toxicity of CyR‐SS‐L to normal COS‐7 cells was significantly lower compared to cancer cells. The cell viability of CyR‐SS‐L was more than 90% on normal COS‐7 cells treated with 1.00 × 10^−6^ m CyR‐SS‐L combined with 808 nm irradiation. Moreover, it is noted that little toxicity of CyR‐SS‐L on normal COS‐7 cells was observed under both dark and light treatment, owing to the lower GSH content in normal cells and a small amount of ROS produced by CyR‐SS‐L combined with irradiation at 808 nm, indicating intelligent selectivity of the PSs. Notably, CyBr‐SS‐L and CyI‐SS‐L exhibited more pronounced PDT effects on A375 and 4T1 cells compared to CyH‐SS‐L, with CyI‐SS‐L demonstrating superior anti‐cancer efficacy against A375 and 4T1 cells compared to the ICG. These findings underscore the intelligent selectivity of CyH‐SS‐L, CyBr‐SS‐L, and CyI‐SS‐L to cancer cells was attributed to CyR‐SG exerting the potent PDT effects.

Next, to assess cell viability, we conducted immunofluorescence staining using Calcein‐AM and PI on 4T1 cells treated with CyR‐SS‐L. Calcein AM penetrates live cells and is hydrolyzed by endogenous esterases to form Calcein, which carries a strong negative charge. Calcein cannot traverse cell membranes and exhibits intense green fluorescence in live cells. In contrast, PI cannot enter live cells and thus remains non‐stained. When cell membranes are compromised and cells undergo death, nucleic acids are exposed and bind to PI, showing intense red fluorescence. As depicted in Figure 2B, only strong green fluorescence was observed in the light‐treated control cells. In contrast, the dark‐treated groups with CyH‐SS‐L, CyBr‐SS‐L, and CyI‐SS‐L exhibited notable green fluorescence along with weaker red fluorescence, indicating minimal dark toxicity of the photosensitizers. However, the light‐irradiated CyH‐SS‐L, CyBr‐SS‐L, and CyI‐SS‐L groups displayed very intense red fluorescence, attributed to the potent PDT effect of CyR‐SG post‐light exposure, resulting in significant tumor cell death. Furthermore, we observed that the ICG group exhibited lower lethality in cancer cells compared to CyH‐SS‐L, CyBr‐SS‐L, and CyI‐SS‐L under identical treatment conditions. These findings were consistent with the MTT results described above (Figure 2A). Finally, we examined the effect of CyH‐SS‐L, CyBr‐SS‐L, and CyI‐SS‐L on the proliferation of KB‐C2 and MCF7/taxol cells overexpressing ABCB1. The resistance fold of KB‐C2 and MCF7/taxol cells for paclitaxel was measured. The resistance fold was calculated by dividing the IC_50_ value for the resistant cell by the IC_50_ for the parental cell. As shown in Figure 2C, KB‐C2 cells had a resistance fold of 215‐fold for paclitaxel with the IC_50_ of 0.28 and 0.0013 × 10^−6^ m on KB‐C2 and KB‐3‐1, respectively. MCF7/taxol cells had a resistance fold of 150‐fold for paclitaxel with the IC_50_ of 0.39 × 10^−6^ and 0.0026 × 10^−6^ m on MCF7/taxol and MCF7, respectively. Subsequently, the efficacy of CyI‐SS‐L, CyBr‐SS‐L, and CyH‐SS‐L photosensitizers on parental cancer cell lines KB‐3‐1, MCF7 and their drug‐resistant sublines KB‐C2 and MCF7/taxol was determined (**Table** **1**). As shown in Figure 2D, the cell survival rate remained above 80% in the absence of light irradiation when the concentration of photosensitizers reached 8 × 10^−6^ m. After irradiation treatment, the IC_50_ of CyI‐SS‐L for all cells was around 1.00 × 10^−6^ m. These data demonstrated that CyI‐SS‐L not only has a remarkable inhibitory effect on parental cancer cells but also ABCB1‐overexpressing MDR cancer cells. In comparison, CyBr‐SS‐L and CyH‐SS‐L had weaker inhibitory activity on KB‐3‐1, KB‐C2, MCF7, and MCF7/taxol cells than CyI‐SS‐L.

**Table 1:** The efficacy of CyR‐SS‐L after irradiation treatment in parental cancer cell lines and drug‐resistant sublines.

Cell line	CyH‐SS‐L^a)^ [× 10^−6^ m]	CyBr‐SS‐L^a)^ [× 10^−6^ m]	CyI‐SS‐L^a)^ [× 10^−6^ m]	Resistance fold of CyI‐SS‐L	Resistance mechanisms
KB‐3‐1	4.64	4.56	1.02	None	Parental
KB‐C2	4.83	4.76	1.01	0.99	ABCB1
MCF7	6.04	4.18	0.95	None	Parental
MCF7/taxol	5.81	4.88	1.00	1.05	ABCB1
4T1	2.30	1.94	0.84	None	None
A375	3.28	2.23	1.00	None	None

^a]^ The values for CyR‐SS‐LRuZ represent the mean concentration of CyR‐SS‐L that is required for IC_50_ (*n* = 3, mean ± SD).

### 2.4 CyR‐SS‐L PSs Have Strong Ability to Produce ROS

**Figure 3:**
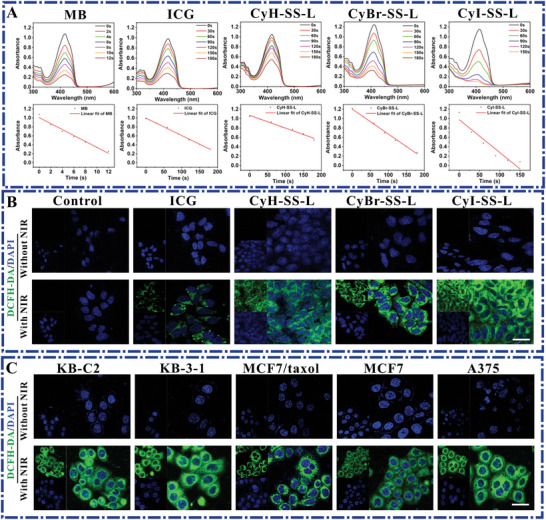
CyH‐SS‐L, CyBr‐SS‐L, and CyI‐SS‐L have a greater ability to generate extracellular ^1^O_2_ than ICG. A) UV absorption was measured in the solution containing 2.5 × 10^−6^ m of CyR‐SS‐L, ICG, or methylene blue B (MB) and GSH (10 × 10^−6^ m) under 200 mW cm^−2^ laser irradiation at 808 nm for different times (0–180 s) using DPBF as ^1^O_2_ collector. B) Measurement of ROS in 4T1 cells treated with CyR‐SS‐L or ICG combined with or without 808 nm light irradiation. C) Determination of ROS in KB‐C2, KB‐3‐1, MCF7/taxol, MCF7, and A375 cells treated with CyI‐SS‐L combined with or without light irradiation (808 nm, 200 mW cm^−2^, 1 min). (DCFH‐DA: *λ*_ex_: 488 nm and *λ*_em_: 500–580 nm). Scale bars: 50 µm.

To further elucidate the generation of ROS, 1,3‐diphenylisobenzofuran (DPBF) was employed as a ^1^O_2_ scavenger to assess ^1^O_2_ production in solution. The results demonstrate that within the CyI‐SS‐L system, DPBF degradation was most rapid, followed by CyBr‐SS‐L and then CyH‐SS‐L, indicating the superior ^1^O_2_ generation capability of CyI‐SS‐L. Additionally, the ^1^O_2_ generation abilities of these three PSs were markedly higher than that of ICG. To quantify ^1^O_2_ production more precisely, using methylene blue (MB) as a ^1^O_2_ standard, experiments yielded ^1^O_2_ quantum yields for ICG, CyH‐SS‐L, CyBr‐SS‐L, and CyI‐SS‐L as 3.10%, 2.140%, 4.02%, and 5.37%, respectively (**Figure** **3**A). Notably, the ^1^O_2_ quantum yields of CyBr‐SS‐L and CyI‐SS‐L were significantly higher than that of ICG. The elevated singlet oxygen generation by CyI‐SS‐L can be attributed to the introduction of heavy atoms, which enhances intersystem crossing (ISC) efficiency between molecular states.

Benefiting from its high‐generation ROS in solution, 2,7‐dichlorodihydrofluorescein diacetate (DCFH‐DA) was employed to detect ROS production in 4T1 cells. Results revealed no observable green fluorescence in the dark control group or the untreated control group, indicating the absence of ROS production in both groups. However, upon addition of CyH‐SS‐L, CyBr‐SS‐L, and CyI‐SS‐L followed by irradiation with an 808 nm laser, strong green fluorescence was observed in the experimental groups, with fluorescence intensity surpassing that of the ICG group (Figure 3B). This indicates the ability of our PSs to generate cytotoxic ROS. To further elucidate the PSs' ROS generation capability, CyI‐SS‐L was selected as the primary photosensitizer, and ROS generation was assessed in four cell lines. Results demonstrated that cells incubated solely with CyI‐SS‐L showed no significant green fluorescence, whereas intense green fluorescence was observed after near‐infrared light irradiation. These experiments underscore the ability of our CyI‐SS‐L to generate cytotoxic ROS in a majority of tumor cells under light exposure.

### 2.5 CyR‐SS‐L PSs Localize Mainly in Mitochondria in Cancer Cells

**Figure 4:**
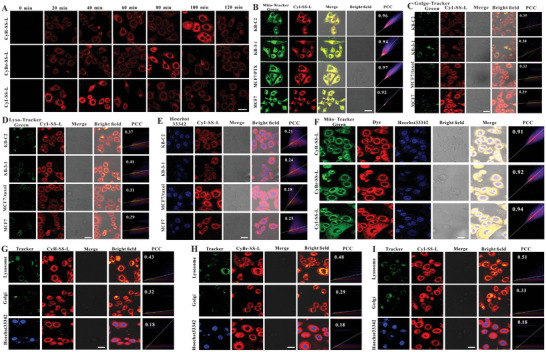
CyR‐SS‐L PSs mainly localize in mitochondria in cancer cells. A) MCF cells were treated with 1 × 10^−6^ m of CyH‐SS‐L, CyBr‐SS‐L, or CyI‐SS‐L and the fluorescence signals generated by the cells at different processing times were collected by CLSM. B–E) The subcellular localization of the PPSs in KB‐C2, KB‐3‐1, MCF7, and MCF7/taxol cells incubated with 1 × 10^−6^ m CyI‐SS‐L for 2 h was detected using CLSM. F) Detection of the mitochondria localization of CyBr‐SS‐L, CyI‐SS‐L and CyH‐SS‐L in 4T1 cells. G–I) Detection of Golgi apparatus, lysosome, and nuclear localization of CyH‐SS‐L, CyBr‐SS‐L, and CyI‐SS‐L, respectively in 4T1 cells. For MitoTracker green, LysoTracker green, and GolgiTracker green: (*λ*_ex_ = 488 nm, *λ*_em_ = 500–580 nm), for nucleus: (*λ*_ex_ = 350 nm, *λ*_em_ = 400–500 nm), for CyR‐SS‐L (*λ*_ex_ = 640 nm, *λ*_em_ = 650–730 nm). Scale bar = 50 µm.

Benefiting from its robust capability in generating ROS, we scrutinized the cellular uptake profiles of three PSs in MCF7 cells over a 0–2 h timeframe. Notably, CyI‐SS‐L exhibited rapid uptake kinetics, reaching its fluorescence peak within 40 min of cell incubation, contrasting with CyH‐SS‐L, which peaked significantly at 100 min, and CyBr‐SS‐L, reaching its zenith around 80 min. This underscores the expedited cellular uptake of our CyI‐SS‐L. Given the cationic nature of the phthalocyanine dye, it aptly targets the negatively charged mitochondrial membrane within cells. We conducted colocalization experiments of CyI‐SS‐L with KB‐C2, KB‐3‐1, MCF7/taxol, and MCF7 cells' mitochondria. Across the four cell lines, CyI‐SS‐L demonstrated robust colocalization with mitochondria, as evidenced by high Pearson's coefficients of 0.96, 0.94, 0.97, and 0.92, respectively (**Figure** **4**B), indicating effective mitochondrial targeting of CyI‐SS‐L and favorable colocalization in both parental and drug‐resistant cancer cells. Furthermore, we assessed CyI‐SS‐L's colocalization with other organelles, revealing notably low colocalization coefficients with the Golgi apparatus, lysosomes, and cell nuclei (Figure 4C–E), underscoring its predominant localization within mitochondria and its role in triggering apoptosis through in situ ROS generation on the mitochondrial membrane. To validate the universality of photosensitizer localization, we coincubated 4T1 cells with 1 × 10^−6^ m concentrations of CyH‐SS‐L, CyBr‐SS‐L, CyI‐SS‐L, and a green fluorescent mitochondrial probe. The fluorescence channels of both entities exhibited significant overlap, with CyI‐SS‐L displaying a colocalization coefficient of 0.94 (Figure 4F). These findings indicate mitochondrial localization of CyR‐SS‐L PSs. Additionally, colocalization coefficients of all PSs with other subcellular organelles were comparably low (Figure 4G–I). The mitochondria targeting of CyR‐SS‐L PSs can be interpreted as that CyR‐SS‐L is a cationic photosensitizer, and the nitrogen cation in its structure can target mitochondria, which typically have a negative charge. It targets the mitochondrial inner membrane through electrostatic attraction between positive and negative charges.

### 2.6 CyI‐SS‐L Combined with Radiation Inhibits ABCB1 and Activates the Mitochondrial Apoptosis Pathway

**Figure 5:**
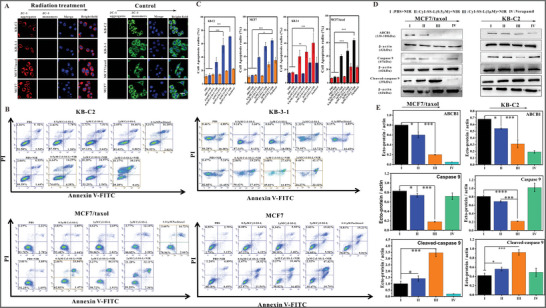
CyI‐SS‐L decreased the mitochondrial membrane potential and induced cell apoptosis in KB‐C2 cells, KB‐3‐1 cells, MCF7 cells, or MCF7/taxol cells through the mitochondrial apoptosis pathway. A) The effect of CyI‐SS‐L on the mitochondrial membrane potential of KB‐C2 cells, KB‐3‐1 cells, MCF7 cells, and MCF7/taxol cells treated with CyI‐SS‐L (1 × 10^−6^ m) combined NIR radiation using JC‐1 probe (*λ*_ex_ = 488 nm, *λ*_em_ = 561 nm). Scale bar = 50 µm. B) The effect of CyI‐SS‐L (1 × 10^−6^ m) on cell apoptosis, analyzed by flow cytometry using membrane‐associated protein V‐FITC and PI in KB‐C2 cells, KB‐3‐1 cells, MCF7 cells, and MCF7/taxol cells under the NIR radiation. C) The bar graph presents the rate of cell apoptosis. D) The effect of CyI‐SS‐L on the expression of ABCB1, Caspase 9, and cleaved‐Caspase 9 proteins by Western blotting in MCF7/taxol cells and KB‐C2 cells. E) Use of densitometry to measure the intensity of the protein band quantified by AI600 images (*p < 0.5, **p < 0.01,***p < 0.001, **** *p* < 0.0001). Scale bars: 50 µm.

We determined the effect of CyI‐SS‐L on mitochondrial membrane potential in KB‐C2, KB‐3‐1, MCF7, and MCF7/taxol cells. The control group showed strong red fluorescence, which indicated that the mitochondrial membrane potential was in a high state, and the cells did not undergo apoptosis. The parental cells and their drug‐resistant sublines treated with the combination of CyI‐SS‐L and radiation showed strong green fluorescence, which indicates that the mitochondrial membrane potential of the cancer cells was in a low state, and the cells were apoptotic (**Figures** **5**A and S15, Supporting Information). Subsequently, the cell apoptosis rate was determined in the parental cells and their drug‐resistant sublines treated with the combination of CyI‐SS‐L and radiation. CyI‐SS‐L (2 × 10^−6^ m) alone caused no significant apoptosis., whereas cells treated with the combination of CyI‐SS‐L and radiation showed significant apoptosis dependent upon the CyI‐SS‐L concentration (Figure 5B,C). Thus, the antiproliferation effect of CyI‐SS‐L combined with radiation on the parental cells and their drug‐resistant sublines was due to inducing cell apoptosis.

This proposed mechanism was verified by Western blotting analysis of MCF7/taxol and KB‐C2 cells. When cells undergo apoptosis, caspase 9 is cleaved into cleaved caspase 9 (Figure 5D,E). In both cell lines treated with the combination of CyI‐SS‐L (0.5 × 10^−6^ or 1 × 10^−6^ m) and radiation, the expression of caspase 9 significantly decreased, while the expression of cleaved caspase 9 increased. Hence, the induction of cell apoptosis by CyI‐SS‐L combined with radiation was caused by activating the mitochondrial apoptosis pathway. Furthermore, the expression of ABCB1 was significantly inhibited in both cell lines treated with CyI‐SS‐L (0.5 × 10^−6^ m or 1 × 10^−6^ m) combined with radiation, comparable to treatment with verapamil (Figure 5D,E). Taken together, the anti‐proliferation effect of CyI‐SS‐L on the ABCB1‐overexpressing MCF7/taxol and KB‐C2 cells results from the inhibition of ABCB1 and induction of the mitochondrial apoptosis pathway.

### 2.7 PDT and Chemotherapy of CyI‐SS‐L Inhibit Tumor Growth in Mice Xenografted with 4T1 Cells

**Figure 6:**
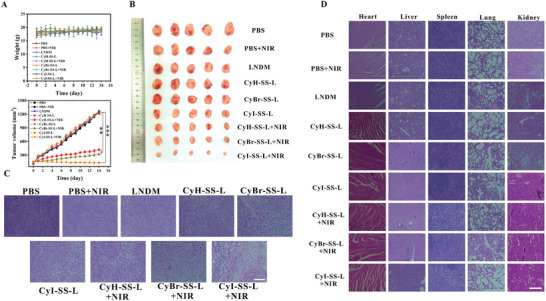
CyBr‐SS‐L, CyI‐SS‐L, and CyH‐SS‐L exhibit remarkable anti‐cancer activity against breast cancer in the 4T1 cell xenograft BALB/c mice model. A) Changes in body weight and tumor volume of mice during the treatment period. B) Images of mouse tumors. C) H&E staining of tumor tissues. D) H&E staining of heart, liver, spleen, lung, and kidney. Scale bars: 200 µm.

To validate the anticancer efficacy of CyH‐SS‐L, CyBr‐SS‐L, and CyI‐SS‐L, we established a tumor model using 4T1 cells. In the PBS and PBS+NIR (irradiation only) groups, the relative tumor volume (*V*/*V*_0_, where *V* and *V*_0_ represent tumor volume at the end and beginning of the experiment, respectively) increased by 7.94‐fold and 7.03‐fold, respectively (**Figure** **6**A). Similarly, in the nonirradiated CyH‐SS‐L, CyBr‐SS‐L, and CyI‐SS‐L groups, tumor volumes increased by 6.05‐fold, 6.32‐fold, and 5.77‐fold, respectively, similar to the 6.62‐fold increase observed in the LNDM group, possibly due to the chemotherapy effect of the SG‐L portion of CyR‐SS‐L post GSH‐induced cleavage. In the irradiated CyH‐SS‐L, CyBr‐SS‐L, and CyI‐SS‐L groups, tumor growth was significantly inhibited, with volumes increasing only by 2.94‐fold, 2.29‐fold, and 0.28‐fold after 14 d, respectively. This effective tumor suppression suggests that a single treatment with CyR‐SH resulted in robust PDT effects post‐NIR radiation and cooperated with chemotherapy to inhibit tumor growth. Tumor images from treated mice confirmed these findings (Figure 6B). To validate the anticancer efficacy of CyH‐SS‐L, CyBr‐SS‐L, and CyI‐SS‐L, we established a tumor model using 4T1 cells. In the PBS group and PBS+NIR group (NIR irradiation only), the relative tumor volume (*V*/*V*_0_, where *V* and *V*_0_ represent the tumor volume at the end and beginning of the experiment, respectively) increased by 7.94‐fold and 7.03‐fold, respectively. Similarly, in the non‐irradiated CyH‐SS‐L, CyBr‐SS‐L, and CyI‐SS‐L groups, the tumor volume increased by 6.05‐fold, 6.32‐fold, and 5.77‐fold, respectively, comparable to the 6.62‐fold increase in the LNDM group, likely due to the chemotherapy effect of the SG‐L portion in CyR‐SS‐L post GSH‐induced cleavage. In the irradiated CyH‐SS‐L, CyBr‐SS‐L, and CyI‐SS‐L groups, tumor growth was significantly inhibited, with volume increases of only 2.94‐fold, 2.29‐fold, and 0.28‐fold, respectively, after 14 d. This effective tumor suppression indicates the excellent PDT effect of CyR‐SH's single treatment post‐NIR radiation, collaborating with chemotherapy to inhibit tumor growth. Tumor images of the treated mice confirmed these results. Throughout the treatment, there were no significant changes in the weight of any group. Furthermore, H&E staining of mouse tumor tissues revealed significant damage in the CyI‐SS‐L+NIR group (Figure 6C). Additionally, no significant damage to the major organs of the mice was observed in the H&E staining, indicating the good biocompatibility and safety of CyH‐SS‐L, CyBr‐SS‐L, and CyI‐SS‐L. CyI‐SS‐L demonstrated outstanding PDT (Figure 6D). Body weight remained stable in all groups throughout the treatment period. Additionally, significant damage was observed in the NIR‐irradiated group. However, no significant damage was noted in the major organs of mice in H&E staining, indicating the excellent biocompatibility of CyH‐SS‐L, CyBr‐SS‐L, and CyI‐SS‐L. CyI‐SS‐L exhibited outstanding PDT.

### 2.8 PDT and Chemotherapy of CyI‐SS‐L Inhibit the Proliferation of Paclitaxel‐Resistant Tumors in Mice Xenografted with MCF7/Taxol Cells

**Figure 7:**
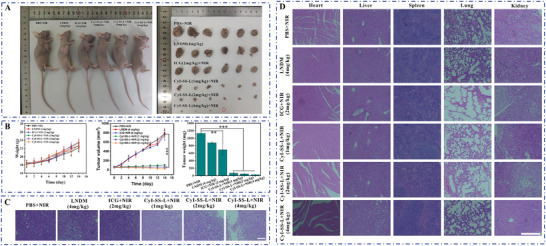
CyI‐SS‐L shows significant anti‐tumor activity against paclitaxel‐resistant breast cancer in the MCF7/taxol cell xenograft BALB/c mice model. A) In vivo imaging of mouse tumors. B) Curves of body weight and tumor volume change during the treatment period. C) H&E staining of tumors from different groups after treatment of 14 d. D) H&E staining of major organs (heart, liver, spleen, lung, and kidney) in mice. (**p < 0.01,*** *p* < 0.001). Scale bars: 200 µm.

Encouraged by the finding that CyI‐SS‐L exhibited much greater inhibition of tumor growth than CyBr‐SS‐L and CyH‐SS‐L in the mice xenografted with 4T1 cells, we used MCF7/taxol cells to establish xenografts in BALB/c female nude mice and determined the efficacy of CyI‐SS‐L (**Figure** **7**A–D). The treatment of CyI‐SS‐L (1 and 2 mg kg^−1^) combined with NIR radiation significantly inhibited tumor growth (inhibition of 93.8% and 95.8%, respectively, calculated based on the tumor weight on Day 14) compared to the LNDM and ICG groups. Furthermore, treatment with CyI‐SS‐L (4 mg kg^−1^) combined with NIR radiation produced a greater inhibition of tumor growth (inhibition of 97.5% on Day 14). Interestingly, the tumor's ablation was observed after two treatments of the CyI‐SS‐L (4 mg kg^−1^) combined with NIR radiation (Figure 7A,B). Moreover, the tumor volume of mice in the CyI‐SS‐L+NIR group was 10× smaller than that in the PBS+NIR group. In addition, during the treatment period, there were no significant differences in the weights of each group of mice, and the damage was to the tumors (Figure 7C), demonstrating the therapeutic effect of CyI‐SS‐L (4 mg kg^−1^) combined NIR radiation. LNDM showed anti‐tumor activity and the SG‐L part of the CyI‐SS‐L played the role of chemotherapy after GSH cleavage. Hence, CyI‐SS‐L combined with NIR radiation has a highly effective anti‐tumor effect via combining PDT and chemotherapy. Additionally, the photosensitizer did not show obvious damage to the important organs of mice in each group, which indicates that the photosensitizer has high biological safety and histocompatibility (Figure 7D).

## 3 Discussion

MDR is a major obstacle to the success of cancer chemotherapy,^[^ ^41^ ^]^ and overcoming MDR remains a challenge in cancer treatment. ABCB1 is one of the major members of the ABC transporters responsible for the development of MDR and has been highly pursued as a potential target to overcome MDR in many types of cancer cells.^[^ ^42^, ^43^ ^]^ Hence, the development of safe and effective ABCB1‐inhibitors has long been a goal to combat MDR. PDT is an approved photo‐activated and non‐invasive medical technique that has been widely used as a treatment for cancer therapy known to lack harmful side effects.^[^ ^44^ ^]^ PDT employs photosensitizers that are preferentially taken up and accumulate in diseased tissues. Photosensitizers are activated by absorption of visible light at an appropriate wavelength which results in the production of ROS such as singlet oxygen, followed by irreversible destruction of tumor tissues. PDT relies on highly toxic ROS produced by photosensitizers. However, the ROS generated from the photosensitizer can be reduced by a high concentration of GSH in cancer cells, which greatly decreases the effect of PDT.^[^ ^45^ ^]^ Depleting intracellular GSH should strengthen the anti‐tumor efficiency of radiotherapy.^[^ ^46^ ^]^ Moreover, PDT can frequently lead to side effects on normal tissues. Therefore, it is very urgent to achieve intelligent selectivity in PDT with the selective killing of tumor tissues without destroying normal tissues.

In this work, to solve the above two problems, we synthesized highly potent CyR‐SS‐L photosensitizers (CyI‐SS‐L, CyBr‐SS‐L and CyH‐SS‐L) having a similar indole ring to ICG. The biggest difference compared to ICG is the presence of LNDM molecules in CyH‐SS‐L. Compared to CyH‐SS‐L, heavy atom Br and I were introduced in CyBr‐SS‐L and CyI‐SS‐L, respectively. Unlike conventional anti‐tumor drugs, CyR‐SS‐L photosensitizers effectively targeted mitochondria and achieved good co‐localization coefficients in drug‐resistant cancer cells, but the CyI‐SS‐L in particular was excellent. Singlet oxygen (^1^O_2_), one of the primary toxic ROS, is generated by energy transfer between the triplet excited state of photosensitizers and the ground state of O_2_. The generation of ^1^O_2_ is one mechanism of production of ROS by photosensitizers.

We found that the ^1^O_2_ producing capacity of CyR‐SS‐L photosensitizers in vitro was stronger than that of ICG. Moreover, unlike traditional photosensitizers, CyR‐SS‐L was capable of generating a large amount of ROS specifically in cancer cells, including drug‐resistant cancer cells, but not in normal cells. It was shown that CyBr‐SS‐L, CyI‐SS‐L, and CyH‐SS‐L generated more ROS during irradiation than ICG in 4T1 cells, and CyI‐SS‐L in particular was potent (Figure 3B). The ROS yield of iodine‐containing CyH‐SS‐L was more than ICG in 4T1 cells, which may be due to the introduction of LNDM with the synergistic effect of photosensitizers.^[^ ^26^, ^27^, ^28^, ^29^, ^30^, ^31^ ^]^ Furthermore, CyI‐SS‐L demonstrated the most efficient ROS production due to the “heavy atom effect”. The process of generation of large amount of ROS by CyR‐SS‐L was explained as follows: CyR‐SS‐L undergoes cleavage yielding CyR‐SG and SG‐L fragments, triggered by the overexpression of glutathione (GSH) within the mitochondria of cancer cells. CyR‐SG, functioning as the photosensitizer component, is employed for PDT. Meanwhile, SG‐L corresponds to LNDM group, a chemotherapy agent with broad‐spectrum tumor cell targeting capabilities. Hence, CyR‐SS‐L was used as a novel and effective anticancer agent by PDT and chemotherapy.

We found that the PDT and chemotherapy using CyR‐SS‐L exhibited a greater inhibition of tumor growth in mice xenografted with 4T1 cells, while CyI‐SS‐L was more potent than CyBr‐SS‐L and CyH‐SS‐L. Of note, CyI‐SS‐L combined with NIR radiation effectively inhibited the proliferation of paclitaxel‐resistant ABCB1‐overexpressing MCF7/taxol cells (IC_50_ = 1.00 × 10^−6^ m, RF = 150). CyI‐SS‐L combined NIR radiation also significantly suppressed the growth of the paclitaxel‐resistant ABCB1‐overexpressing KB‐C2 cells (IC_50_ = 1.01 × 10^−6^ m, RF = 215). Moreover, CyI‐SS‐L combined with NIR radiation was efficacious in inhibiting the proliferation of paclitaxel‐resistant tumors in mice xenografted with MCF7/taxol cells, where inhibition of 97.5% of cells was found on Day 14 and tumors were completely ablated. The inhibitory activity CyI‐SS‐L of against the MDR cancer tumor is much higher than that of ICG, which originated from the “heavy atom effect” and the introduction of LNDM. Notably, The PDT and chemotherapy of CyI‐SS‐L have high biological safety and histocompatibility due to the selectivity of PDT on tumor tissues.

Interestingly, when the cells were treated with the mitochondrial‐targeted CyR‐SS‐L, GSH was successfully bound to the disulfide bond on the CyR‐SS‐L and its content was significantly reduced by photosensitizers. The depletion of GSH was observed in KB‐3‐1, KB‐C2, MCF7 and MCF7/taxol cells treated with CyI‐SS‐L combined with NIR radiation (Figure 3B). Thus, CyR‐SS‐L not only exerts the PDT by generating ROS but also by depleting GSH.

ROS, mainly produced by the mitochondria, can drive cell death by apoptosis.^[^ ^47^ ^]^ Moreover, mitochondrial‐mediated apoptosis is associated with loss of mitochondrial membrane potential.^[^ ^48^ ^]^ Hence, ROS resulting in apoptosis can be evidenced by reduced mitochondrial membrane potential. The present study demonstrated that photosensitizers were mainly localized on mitochondria and ROS was generated. Additionally, we showed that mitochondrial membrane potential was decreased in the KB‐C2, KB‐3‐1, MCF7, and MCF7/taxol cells treated with mitochondrial‐targeted CyI‐SS‐L combined with NIR radiation. Further, the combination treatment of CyI‐SS‐L and NIR radiation induced the apoptosis of KB‐C2, KB‐3‐1, MCF7, and MCF7/taxol cells, as shown by flow cytometry analysis. The combination treatment of CyI‐SS‐L and NIR radiation significantly up‐regulated cleaved caspase‐3 protein while down‐regulated protein level as compared to control.^[^ ^49^ ^]^ Our data suggest the combination treatment of CyI‐SS‐L and NIR radiation induced ROS‐dependent caspase‐3‐mediated mitochondrial intrinsic apoptotic pathway and was responsible for the decreased proliferation.

Considering that ABCB1, a drug‐efflux transporter, is overexpressed in KB‐C2 and MCF7/taxol cells, we investigated the effect of CyI‐SS‐L on the expression of ABCB1 by Western blotting. CyI‐SS‐L significantly suppressed the expression of ABCB1 compared to control in KB‐C2 and MCF7/taxol cells under NIR radiation. Considering that overexpression of ABCB1 in drug‐resistant cells is associated with a decrease in intracellular ROS levels, and increasing ROS levels can effectively inhibit the expression of ABCB1.^[^ ^50^, ^51^ ^]^ Hence, the inhibition of ABCB1 by CyI‐SS‐L may be originated from the production of high levels of ROS. Therefore, CyI‐SS‐L combined with radiation inhibited the anti‐proliferation of ABCB1‐overexpressing drug‐resistant cells in vitro and in vivo by inhibition of ABCB1 and activation of the mitochondrial apoptosis pathway mediated by generation of a large amount of ROS.

## 4 Conclusion

We synthesized three near‐infrared light‐responsive photosensitizer‐drug conjugates CyR‐SS‐L, in which the introduction of iodine and bromine greatly enhanced the ROS production capacity of the CyR‐SS‐L, which enhanced PDT. Furthermore, a large amount of endogenous GSH in cancer cells was consumed by CyR‐SS‐L, which further enhanced the effect of PDT. Interestingly, CyR‐SS‐L was more efficacious in inhibiting the proliferation of paclitaxel‐resistant breast cancer tumors than ICG by inhibition of ABCB1 and activating the mitochondrial apoptosis pathway with very little toxicity in normal cells due to the intelligent selectivity of PDT. These results suggested they can be extrapolated to use in humans. Thus, CyR‐SS‐L may be employed as an excellent PDT agent to target tumors without obviously damaging healthy tissue and represent an efficacious treatment for MDR cancers by combined PDT, GSH depletion, and chemotherapy.

## 5 Experimental Section

### Reagents and Equipment

4‐Bromophenylhydrazine hydrochloride, (4‐Iodophenyl) hydrazine hydrochloride, cyclohexanone, cycloamine dihydrochloride, lonidamine, 1,3‐diphenylisobenzofuran (DPBF), ICG, and methylene blue B (MB) were purchased from Aladdin (Shanghai, China). DMEM basic medium, RPMI‐1640 basic medium, special grade fetal bovine serum (FBS), and penicillin‐streptomycin solution (100×) were purchased from Pricella (Wuhan, China). MitoTracker Green (C1048), GSH and GSSG assay kit (S0053), LysoTracker Green (C1047S), GolgiTracker Green (C1045S), Hoechst 33342 (C1027), thiazolyl blue tetrazolium bromide (MTT), Calcein/propidium iodide (PI) cell viability (C2015L), enhanced mitochondrial membrane potential assay kit with JC‐1 (C2003S), annexin V‐FITC apoptosis detection kit (C1062L), reactive oxygen species assay kit (ROS assay kit, S0033S), cell lysis buffer (P0013), and anti‐β‐actin antibody (AA128) were purchased from Beyotime Biotechnology (Shanghai, China). Paclitaxel (P106869) and verapamil (V303890) were obtained from Aladdin (Shanghai, China). MDR1/ABCB1 (#E1Y7B) rabbit mAb (#13342S) and Caspase‐9 (C9) mouse mAb (#19526T) were purchased from Cell Signaling Technology (Beverly, MA, United States).

The UV absorption spectrum of the photosensitizer was obtained on a UV‐2700i UV spectrophotometer, purchased from Japan Instruments Co., Ltd (Shanghai, China). The fluorescence emission spectrum was tested on the F‐7000 fluorescence spectrophotometer (Shimadzu Co., Ltd., Japan). All fluorescence imaging tests were conducted using TCS SP8 laser confocal microscopy (Leica, Germany).

### Cell Lines and Cell Culture

The human carcinoma cell line KB‐3‐1 and its colchicine‐selected multidrug‐resistant KB‐C2 cell line overexpressing ABCB1 were kindly provided by Dr. Shin‐ich Akiyama. The human breast cancer cell line MCF7 and its paclitaxel (taxol) selected multidrug‐resistant MCF7/taxol cell line overexpressing ABCB1 were purchased from Suzhou Qianshe Biotechnology Co., Ltd (Suzhou, China). In addition, the mouse breast cancer cell line 4T1, human malignant melanoma cell line A375 cells, and the African green monkey kidney COS‐7 cell line were purchased from the Institute of Basic Medicine (IBMS), Chinese Academy of Medical Sciences (Beijing, China). The 4T1, MCF7, and MCF7/taxol cells were cultured in RPMI‐1640 medium supplemented with 10% fetal bovine serum and 1% penicillin‐streptomycin. COS‐7, A375, KB‐3‐1, and KB‐C2 cells were cultured in DMEM supplemented with 10% fetal bovine serum and 1% penicillin‐streptomycin.

### Synthesis and Characterization of CyR‐SS‐L

CyR‐SS‐L was synthesized according to the route outlined in Scheme S1 (Supporting Information). The intermediate and final products were completely characterized by ^1^H NMR, ^13^C NMR, and high‐resolution mass spectrometry (HRMS), shown in Figures S1–S9 (Supporting Information).

### Determination of Optical Properties of CyR‐SS‐L

Stock solutions of CyBr‐SS‐L, CyI‐SS‐L, or CyH‐SS‐L (10 × 10^−3^ m) were prepared in DMSO and were further diluted in methanol (MeOH), dichloromethane (DCM), dimethyl sulfoxide (DMSO), ethyl alcohol (EtOH), ethyl acrylate (EA), or N, N‐dimethylformamide (DMF) to make 5 × 10^−6^ m working solutions. Finally, the UV–visible absorption spectra of the three compounds in six solvents were determined using a UV–visible spectrophotometer.

### Determination of the Cleavage of CyR‐SS‐L by GSH

To determine the cleavage of CyR‐SS‐L by GSH, stock solutions of CyBr‐SS‐L, CyI‐SS‐L, or CyH‐SS‐L in DMSO (5 × 10^−6^ m) were mixed with 500 µL of 10 × 10^−3^ m GSH and incubated in a 37 °C water bath for 30 min. After incubation, the UV–visible absorption spectrum of the compound was measured using a UV–visible spectrophotometer. In addition, the solution of CyR‐SS‐L mixed with GSH was detected by mass spectroscopy.

### Detection of the Content of Intracellular GSH in MDR Cancer Cells

KB‐3‐1, KB‐C2, MCF7, and MCF7/taxol cells were treated with CyI‐SS‐L for 1 h, then irradiated at 808 nm (200 mW cm^−2^) for 1 min. After incubation for another 24 h, cells were washed with cold PBS, incubated with Protein Removal Buffer, repeatedly frozen in liquid nitrogen and thawed in a 37 °C water bath two times. The samples were kept in an ice bath for 5 min and centrifuged at 10 000 rpm for 10 min. Levels of oxidized glutathione (GSSG) and GSH were measured using the GSSG and GSH assay kit.

### Detection of the Yield of Extracellular Singlet Oxygen

The ability of compounds dissolved in DMSO to produce singlet oxygen in vitro was detected by measuring the change in absorption intensity of DPBF at 418 nm. A mixed solution containing 2.5 × 10^−6^ m of CyR‐SS‐L, ICG, or methylene blue B (MB) and GSH (10 × 10^−6^ m) was prepared and then incubated at 37 °C for 0.5 h. DPBF (20 × 10^−6^ m) was added after the incubation was completed, and UV absorption was measured after 200 mW cm^−2^ laser irradiation at 808 nm for a specific time. The detection interval was 30 s. The yield of singlet oxygen *Φ_Δ_* was calculated using the following formula: *Φ_Δ_ = Φ*_(MB)_ *× k*_(ps)_ *× F*_(MB)_ */ k*_(MB)_ *× F*_(ps)_., where *K* is the slope of the absorbance of DPBF at 418 nm, and *F* is the correction factor of absorption, which can be determined by the formula *F* = 1–10^−OD^ (where OD represented the absorbance of the photosensitizer at 808 nm and methylene blue at 660 nm), *Φ*_MB_ represented the singlet state oxygen quantum yield of MB.

### Detection of the Production of Intracellular ROS

The singlet oxygen indicator 2′,7′‐dihydrodichlorofluorescein diacetate (DCFH‐DA) was used to detect intracellular ROS production. Cells (1 × 10^4^ per mL) were seeded into glass cell culture dishes and cultured for 24 h. Cells were then treated with 1 × 10^−6^ m of CyH‐SS‐L, CyBr‐SS‐L, CyI‐SS‐L, or ICG for 12 h and incubated with 10 × 10^−3^ m of DCFH‐DA for another 20 min. The dark group did not receive light radiation, the control group was not treated with the indicated compounds, and the light group was irradiated at 808 nm (200 mW cm^−2^) for 1 min. Finally, cellular imaging was observed under an inverted fluorescence microscope.

### In Vitro Cell Drug Uptake Studies

Cells were seeded at a density of 5 × 10^4^ cells per well in a six‐well plate. After 24 h of culture, CyBr‐SS‐L, CyI‐SS‐L, or CyH‐SS‐L (1 × 10^−6^ m) was diluted with medium and added to plates. After the specified incubation time, the supernatant was cleared, the cells were treated and fixed with 4% paraformaldehyde, washed with PBS and analyzed with a confocal laser scanning microscope (CLSM).

### Subcellular Colocalization Analysis

Cells were seeded in glass petri dishes at a density of 1 × 10^4^ per mL and cultured for 24 h. Then cells were treated with 1 × 10^−6^ m CyBr‐SS‐L, CyI‐SS‐L, or CyH‐SS‐L for 12 h. Cells were stained with commercial organelle dyes and visualized with CLSM according to the experimental method of the subcellular colocalization kit.

### MTT Assay

The cytotoxicity effect of CyBr‐SS‐L, CyI‐SS‐L, CyH‐SS‐L, ICG, or LNDM in KB‐3‐1, KB‐C2, MCF7, MCF7/taxol, 4T1, A375 cells, and COS‐7 cells was detected by an MTT assay. Cells (2 × 10^5^/100 µL) were seeded into 96‐well plates and cultured for 24 h. The dark group was continuously incubated for 24 h, and the light group was irradiated with 808 nm laser (200 mW cm^−2^) for 1 min after 12 hours’ treatment of compounds and continued to incubate for 12 h after irradiation. The old medium containing the compound was removed and 100 µL of fresh medium containing MTT was added to each well. The medium was removed after incubation for 4 hours, then 200 µL of DMSO was added to each well and plates were placed on a shaking table for 0.5 h to fully dissolve the crystal. At 490 and 630 nm, the absorbance of each well was measured with a SpectraMAX i3x multifunctional microplate reader (Molecular Devices, CA, USA). and cell viability was calculated. The formula for calculating cell viability is as follows: cell viability (%) = (OD_experiment 570 –_ OD_experiment 630_) / (OD_control 570 –_ OD _control 630_) × 100%.

### Cell Viability Imaging Studies

Calcein AM and propidium iodide (PI) were used to perform live and dead cell staining experiments. 4T1 cells (1 × 10^4^ per mL) were inoculated into glass cell culture dishes and cultured for 24 h. Cells were then treated with 1 × 10^−6^ m CyBr‐SS‐L, CyI‐SS‐L or CyH‐SS‐L. After 12 h, cells in the light group were irradiated with an 808 nm laser for 1 min, and cultured for another 12 h. The old medium was removed and washed with PBS, 1 µL Calcein‐ AM and PI were added to each well, incubated for 30 min, and then observed under the inverted fluorescence microscope.

### Cell Apoptotic Rate Analysis

Cells (1 × 10^4^ per mL) were seeded in glass cell culture dishes and cultured for 24 h. The cells were treated with 1 × 10^−6^ m CyBr‐SS‐L, CyI‐SS‐L and CyH‐SS‐L, respectively. After 12 h, the light group was irradiated with laser light and the dark group was not treated. Annexin V‐FITC (5 µL) and PI (5 µL) were added after the incubation was continued for 6 hours. After 20 min of culture, the stained cells were immediately detected by Beckman‐Coulter DXFLEX flow cytometry.

### Detection of Mitochondrial Membrane Potential

Cells were inoculated into a laser confocal Petri dish and incubated at constant temperature for 24 h. Cells were treated with CyI‐SS‐L (1 × 10^−6^ m) for 2 h and irradiated at a wavelength of 808 nm (200 mW cm^−2^, 1 min), followed by incubation for 10 h. The no‐light irradiation group was continued for 12 h. After washing three times with cold PBS, cells were incubated with JC‐1. After washing with cold PBS, cells were observed using confocal microscopy.

### Western Blotting Analysis

KBC2 or MCF7/taxol cells treated with CyI‐SS‐L were collected and washed twice with ice‐cold PBS and lysed with cold radioimmune precipitation assay (RIPA) buffer for 30 min on ice. The lysates were centrifuged at 15 000 rpm for 10 min at 4 °C and the supernatant was collected for analysis. The protein concentration was determined using a BCA protein assay reagent kit. The protein samples were separated on SDS‐polyacrylamide gel electrophoresis and transferred onto PVDF membranes in an ice bath. The membrane was submerged in 5% skim milk for 2 h, washed, and incubated with the primary antibody at 4 °C overnight. After washing with TBST (tris‐buffered saline/0.05% Tween 20), the membrane was incubated with the secondary antibody. Finally, the membrane was washed with TBST and visualized by an enhanced chemiluminescence detection system (GE Healthcare).

### In Vivo Antitumor Efficacy of CyBr‐SS‐L, CyI‐SS‐L, and CyH‐SS‐L in BALB/c Mice with 4T1 Breast Cancer Cell Xenografts

Female BALB/c mice at a weight of 12–15 g were obtained from Beijing Vital River Laboratory Animal Technology Co. Ltd. All handling of experimental animals was in accordance with the guidelines for laboratory animals established by the Animal Care and Use Committee of Weifang Medical University (Certificate Number 2021SDL328). A tumor model was established using mouse breast cancer cells (4T1 cells) inoculated under the left armpit of the mice. When the volume of 4T1 tumors reached 200 mm^3^, mice were divided into nine groups (*n* = 5). The first group was treated with 100 µL of PBS; the second group was treated with 100 µL PBS for 12 h and received 808 nm (200 mW cm^−2^, 5 min) of light radiation; the third, fifth, and seventh groups were treated with 100 µL of CyBr‐SS‐L, CyI‐SS‐L or CyH‐SS‐L (1 mg kg^−1^), respectively. The fourth, sixth, and eighth groups were treated with 100 µL of CyBr‐SS‐L, CyI‐SS‐L or CyH‐SS‐L (1 mg kg^−1^) for 12 h and then received 808 nm (200 mW cm^−2^, 5 min) radiation; the ninth group was treated with 100 µL of LNDM. In all groups, PBS and compounds were injected intratumorally while mice were under anesthesia. Mice body weight and tumor volume were measured daily until day 14. The mice were sacrificed, and the main organs and tumors were isolated and stained with H&E.

### In Vivo Antitumor Efficacy of CyI‐SS‐L in Nude Mice with MDR Human Breast Cancer Cell (MCF7/taxol) Transplanted Tumor

BALB/c female nude mice weighing 16–20 g were obtained from Beijing Weitahe Experimental Animal Technology Co., Ltd. All animal experiments were conducted in accordance with the guidelines for experimental animals formulated by the Animal Care and Use Committee of Weifang Medical University (certificate No. 2021SDL328). BALB/c mice were inoculated with MCF7/taxol cells in their left armpit. When the volume of tumor reached 50 mm^3^, the mice were divided into six groups (*n* = 5): PBS+ NIR group; LNDM (2 mg kg^−1^) group; ICG (2 mg kg^−1^) + NIR group; CyI‐SS‐L (1 mg kg^−1^) + NIR group; CyI‐SS‐L (2 mg kg^−1^) + NIR group; and CyI‐SS‐L (4 mg kg^−1^) + NIR group. Illumination using an 808 nm laser (200 mW cm^−2^, 15 min) was used to irradiate the tumor site in mice treated with the indicated dose of PBS, ICG, or CyI‐SS‐L two times (once a week) in all NIR groups. In all groups, PBS and compounds were injected into the tumor of mice with anesthesia. The body weight and tumor volume of the mice were measured the next day and recorded until the 14th day. The mice were sacrificed, and the main organs and tumors were fixed and embedded in paraffin, then sectioned, and stained with H&E.

### Statistical Analysis

All experiments were repeated at least three times and results were expressed as mean ± SD. The differences were determined by using the two‐tailed Student's t‐test. The significance was determined at *p* < 0.05.

## Conflict of Interest

The authors declare no conflict of interest.

## Supporting information

Supporting Information

## Data Availability

The data that support the findings of this study are available in the supplementary material of this article.
