# Supplementary material for: Glutathione‐Sensitive Photosensitizer–Drug Conjugates Target the Mitochondria to Overcome Multi‐Drug Resistance in Cancer
Source: Adv Sci (Weinh). 2024 Jun 19;11(30):2307765. doi: 10.1002/advs.202307765 (PMC11321625; doi:10.1002/advs.202307765)
Supplement: Supplementary file 1 — Supporting Information [file ADVS-11-2307765-s001.docx]

Supporting Information

Title (Glutathione-sensitive photosensitizer-drug conjugates target the mitochondria to overcome multi-drug resistance in cancer)

*Weiguo Song^#^, Hekai Yang^#^, Ying Wang^#^, Shuzhen Chen, Wenda Zhong*, Qian Wang, Wenshuo Ding, Guangzhao Xu, Chen Meng, Ying Liang, Zhe-Sheng Chen, Shuhua Cao*, Liuya Wei*, Fahui Li **

**Table of Contents**

**Scheme S1.** Synthetic route of CyR-SS-L **39**

[**Figure S1.** ESI-HRMS of CyBr-SS-L **40**](#_Toc12882)

[**Figure S2.** ^1^H NMR of CyBr-SS-L](#_Toc22945) **40**

[**Figure S3.** ^13^C NMR of CyBr-SS-L](#_Toc12863) **41**

[**Figure S4.** ESI-HRMS of CyI-SS-L](#_Toc24442) **41**

[**Figure S5.** ^1^H NMR of CyI-SS-L **4**](#_Toc2680)**2**

[**Figure S6.** ^13^C NMR of CyI-SS-L **4**](#_Toc10653)**2**

[**Figure S7.** ESI-HRMS of CyH-SS-L **4**](#_Toc832)**3**

[**Figure S8.** ^1^H NMR of CyH-SS-L **4**](#_Toc19153)**3**

[**Figure S9.** ^13^C NMR of CyH-SS-L **4**](#_Toc18854)**4**

**[Figure S10.](#_Toc5053)** [The proposed reactions for CyR-SS-L in the GSH solution](#_Toc5053)**[....................44](#_Toc5053)**

[**Figure S11.** ESI-HRMS of CyBr-SS-L in the GSH solution **4**](#_Toc24492)**5**

[**Figure S12.** ESI-HRMS of CyI-SS-L in the GSH solution **4**](#_Toc24492)**5**

[**Figure S13.** ESI-HRMS of CyH-SS-L in the GSH solution **4**](#_Toc24492)**6**

[**Figure S14.** Photodegradation curves of DPBF in the presence of ICG **4**](#_Toc32584)**6**

**Figure S15.** The mitochondrial membrane potential was detected using flow cytometry

**......................................................................................................................................47**

**Table S1.** Singlet oxygen yield (*Φ_△_*) of MB, ICG, CyR-SS-L**...................................48**

**Scheme S1 Synthesis of CyR-SS-L.** Reagents and conditions: (a) CH_3_COOH**;** (b) *o*-dichlorobenzene, 84% for **3a**, 69% for **3b**, 79% for **3c**; (c) POCl_3_, DMF, 65%; (d) Ac_2_O, AcONa, 52% for **6a**, 45% for **6b**, 41% for **6c**; (e) Et_3_N, CH_3_OH, DMF; (f) EDC, DMAP, CH_2_Cl_2_, 12% for **CyBr-SS-L**, 9% for **CyI-SS-L**, 11% for **CyH-SS-L.**


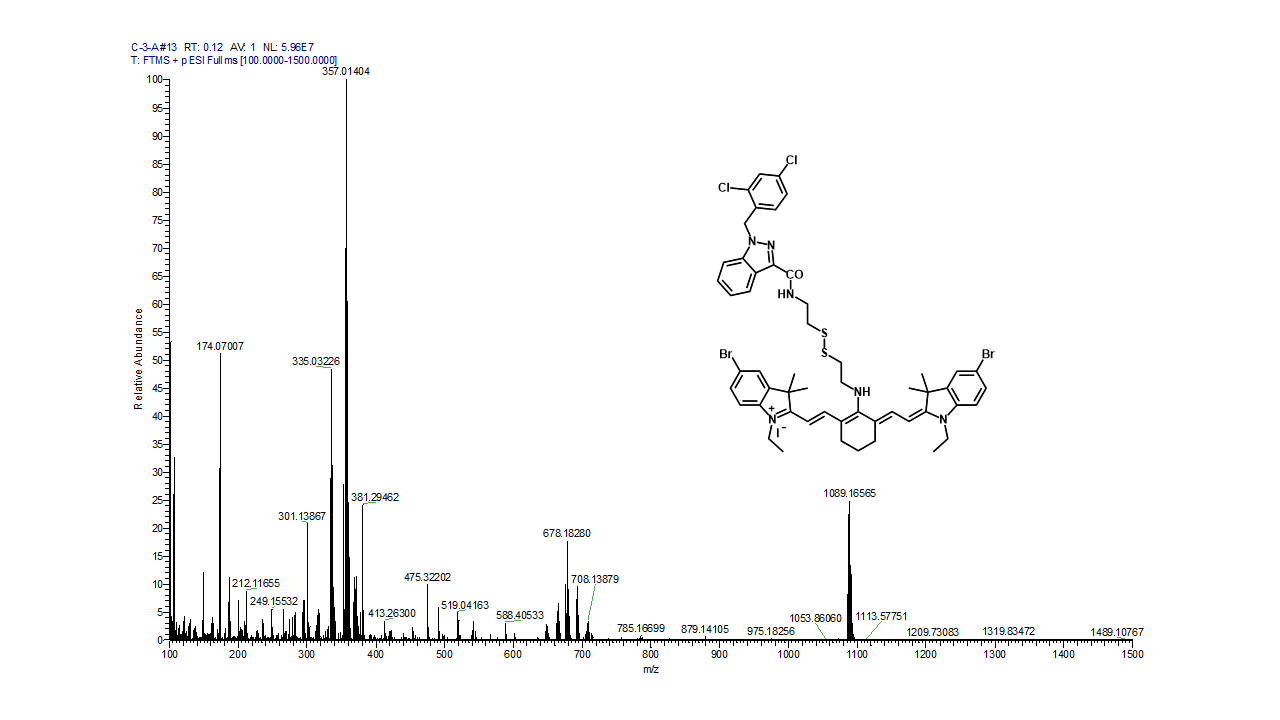


**Figure S1** ESI-HRMS of CyBr-SS-L


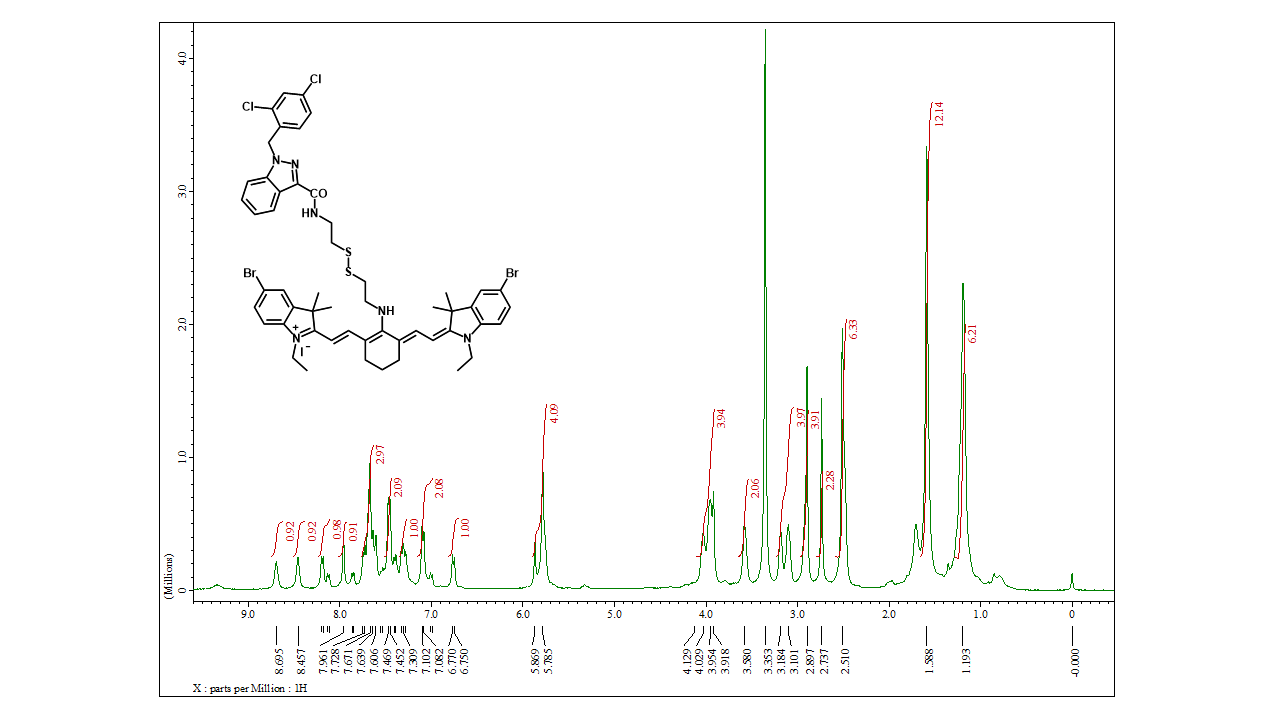


**Figure S2** ^1^H NMR of CyBr-SS-L


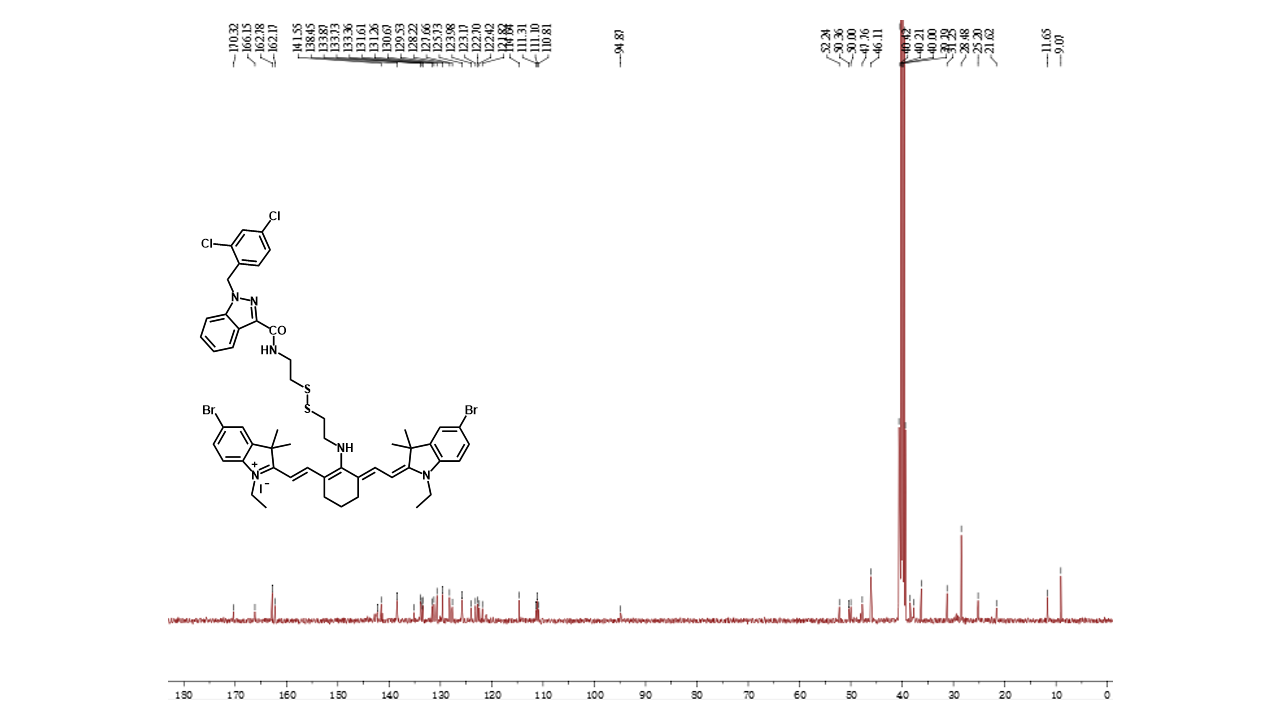


**Figure S3** ^13^C NMR of CyBr-SS-L


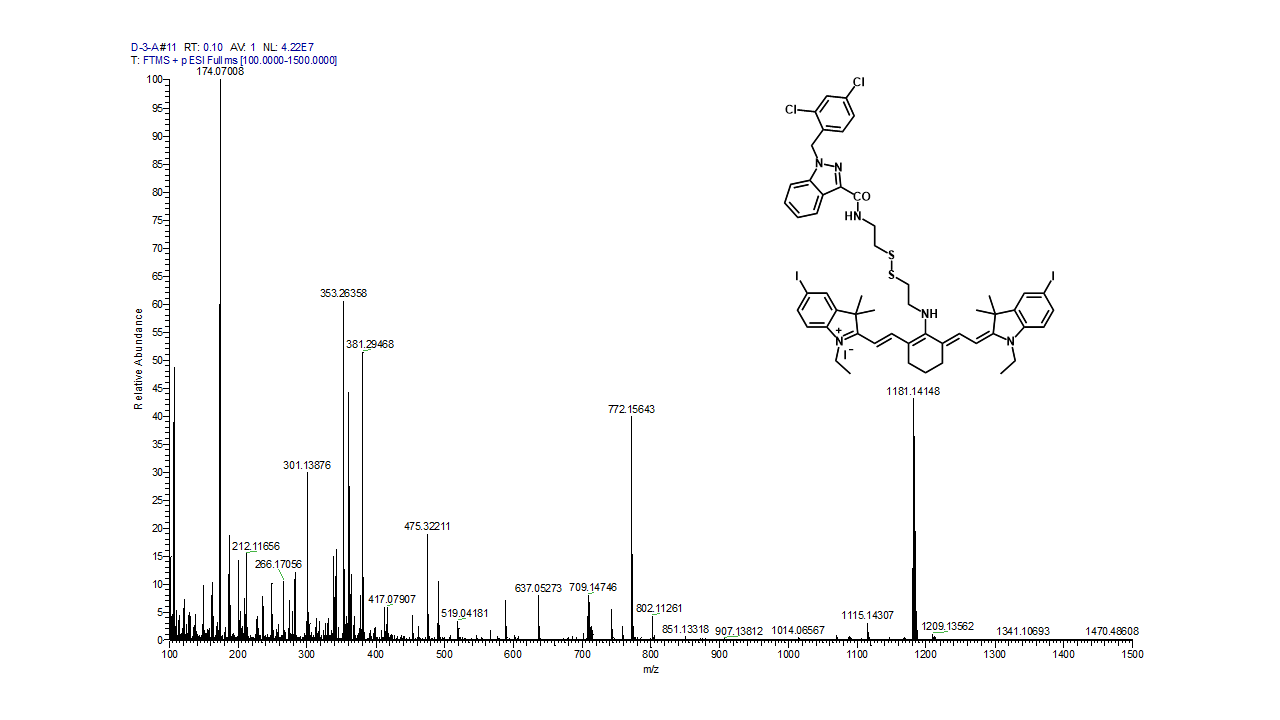


**Figure S4** ESI-HRMS of CyI-SS-L


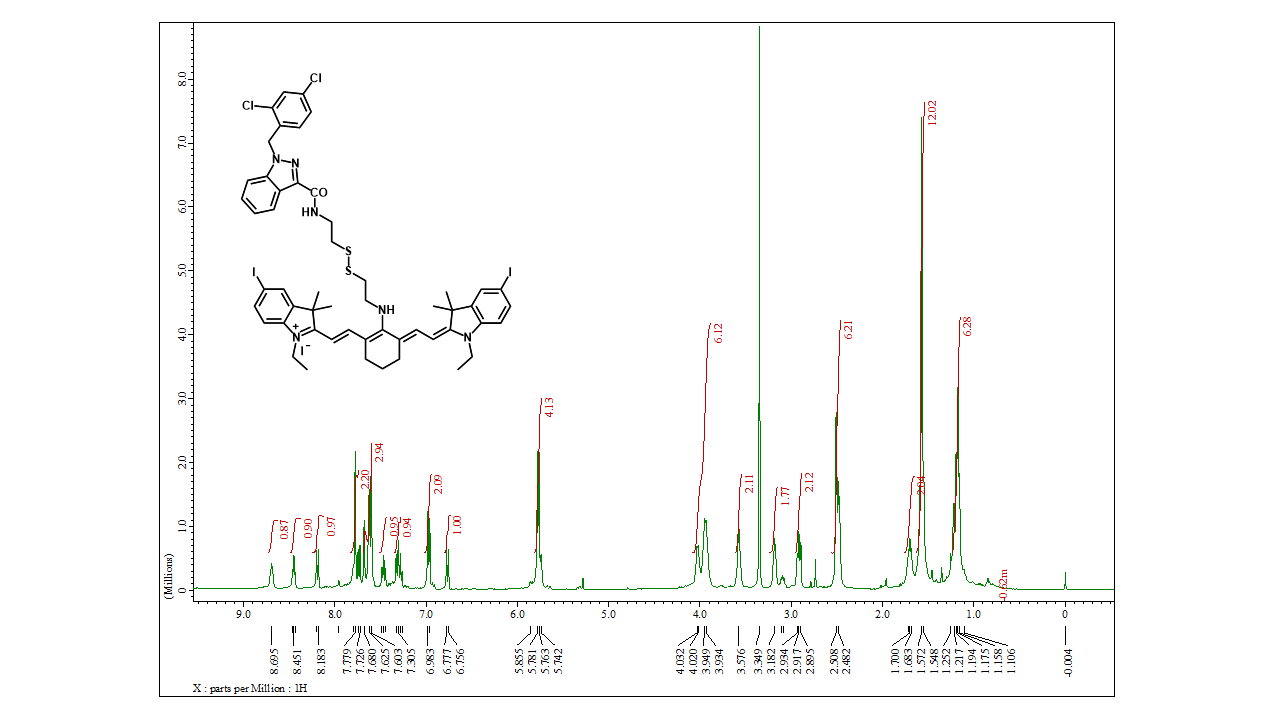


**Figure S5** ^1^H NMR of CyI-SS-L


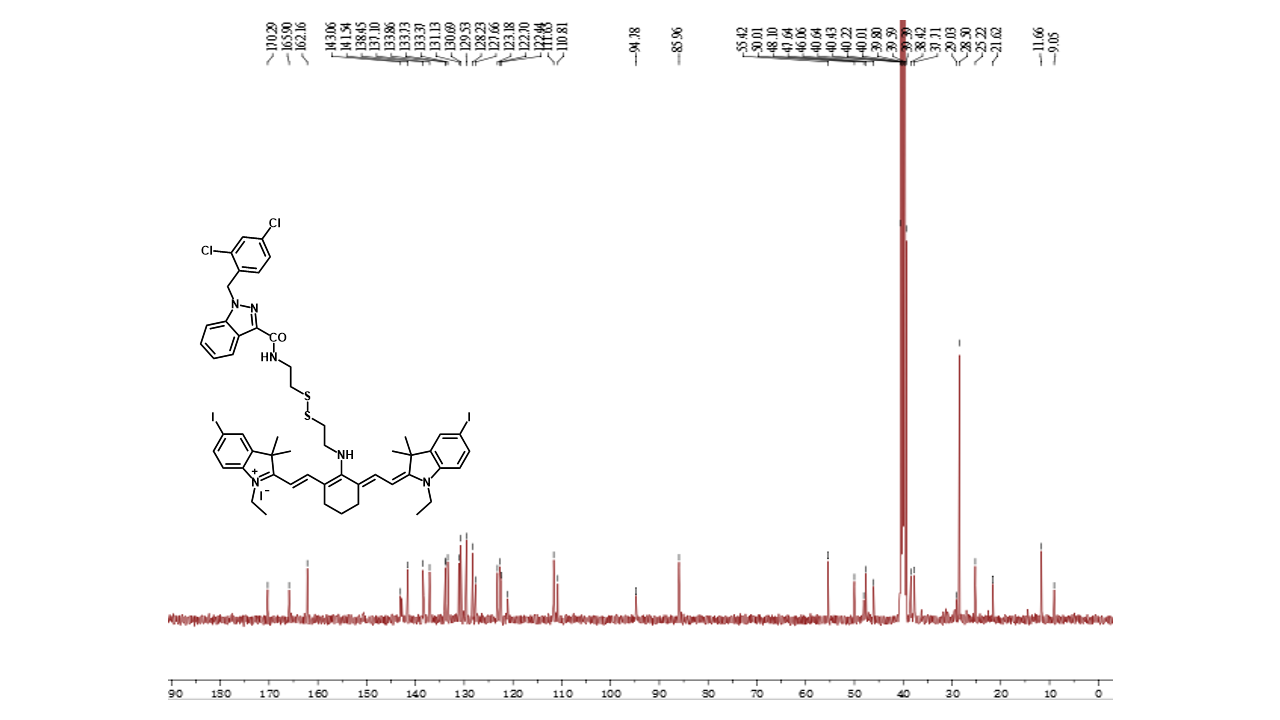


**Figure S6** ^13^C NMR of CyI-SS-L


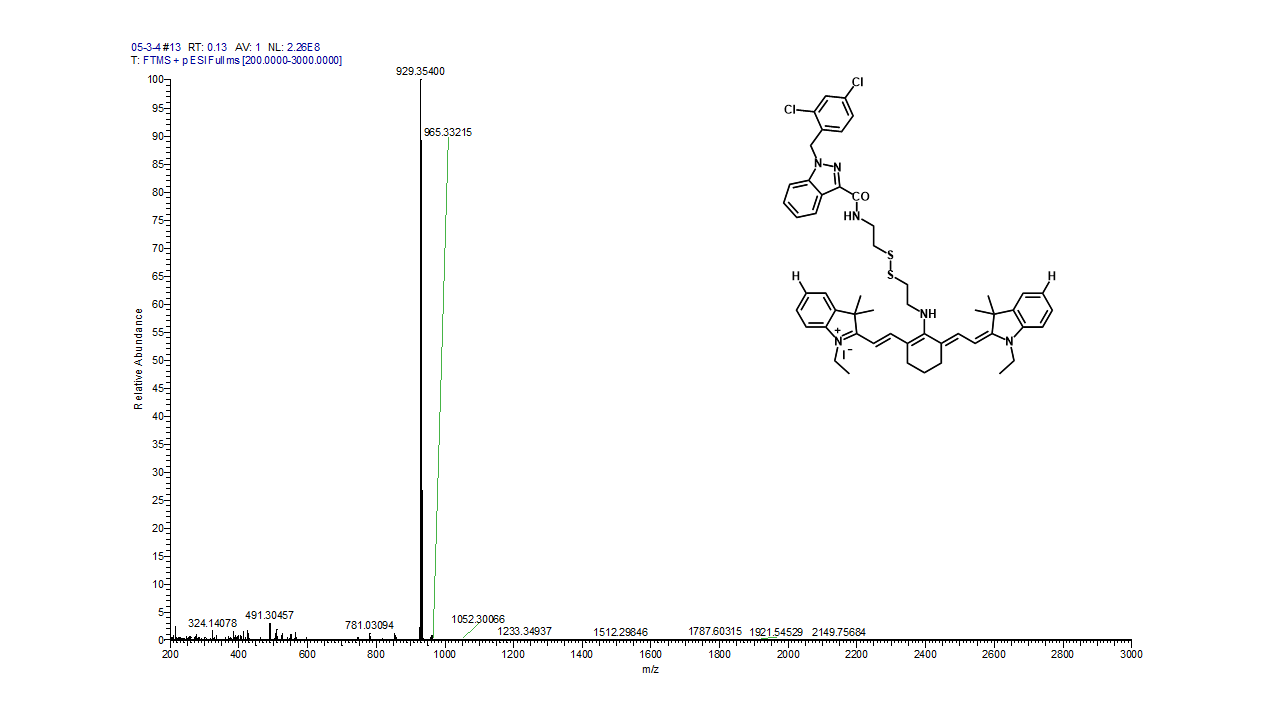


**Figure S7** ESI-HRMS of CyH-SS-L


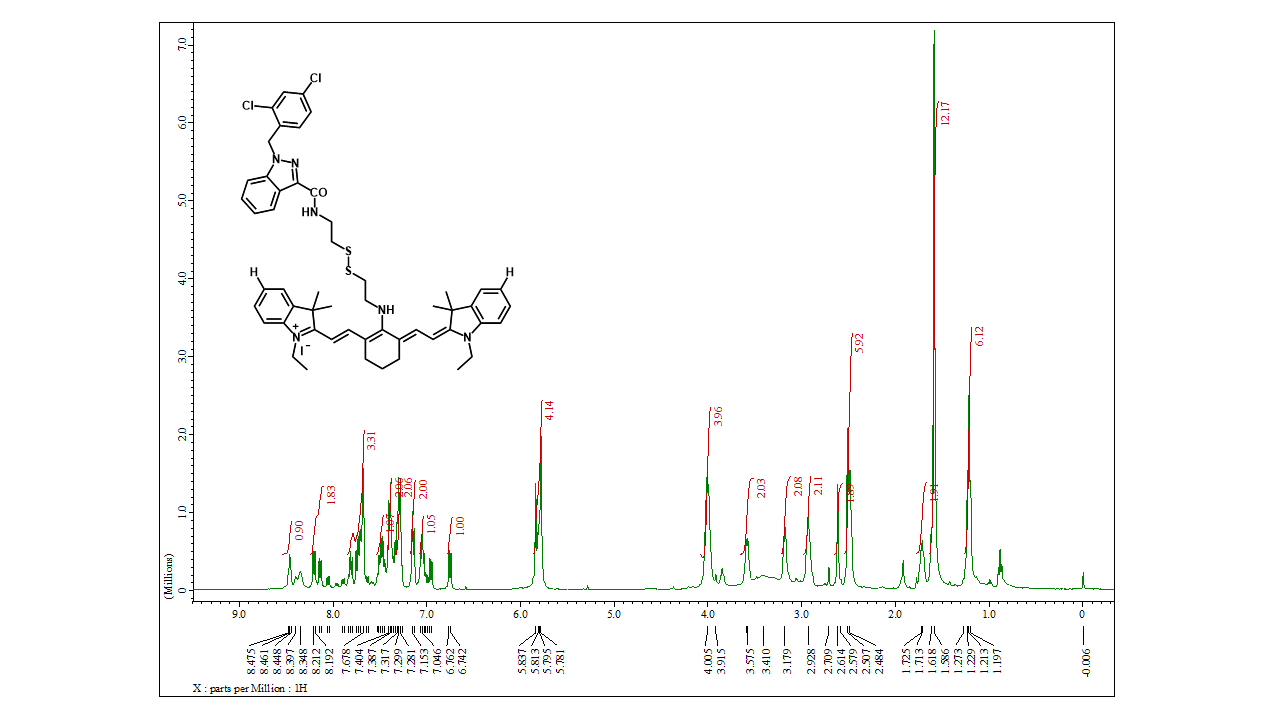


**Figure S8** ^1^H NMR of CyH-SS-L


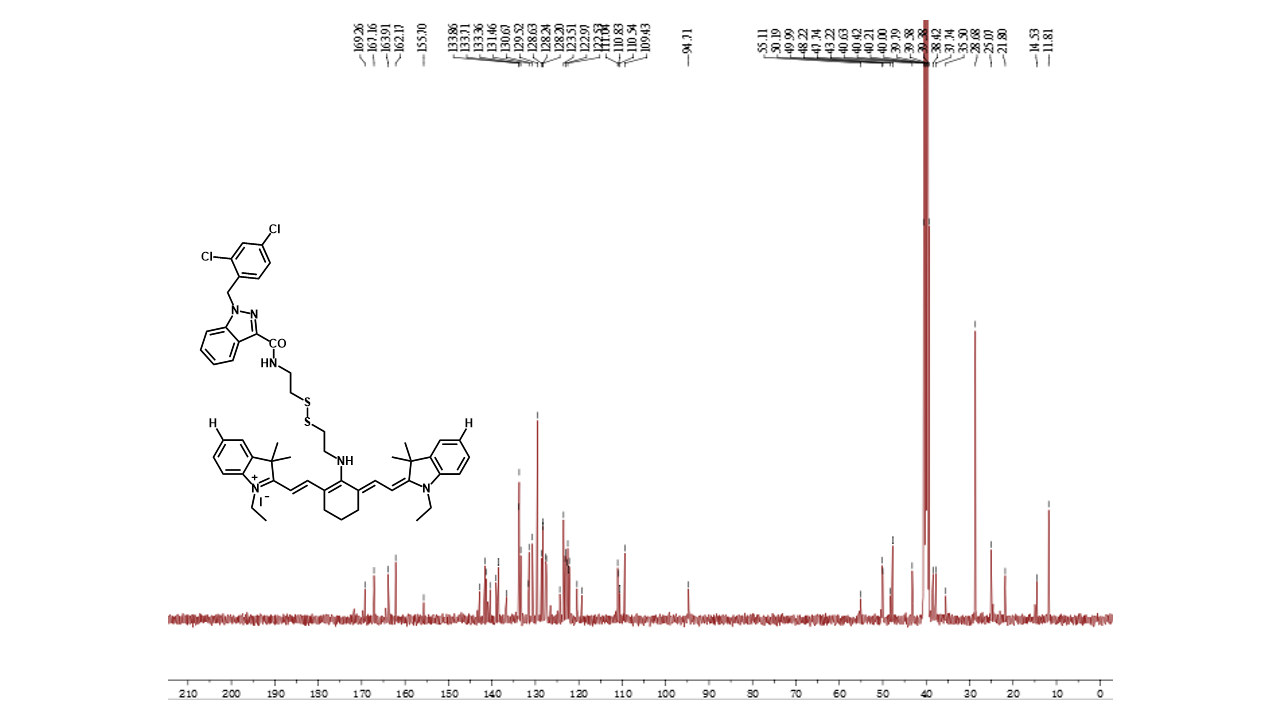


**Figure S9** ^13^C NMR of CyH-SS-L

**Figure S10** The proposed reactions for CyR-SS-L in the GSH solution


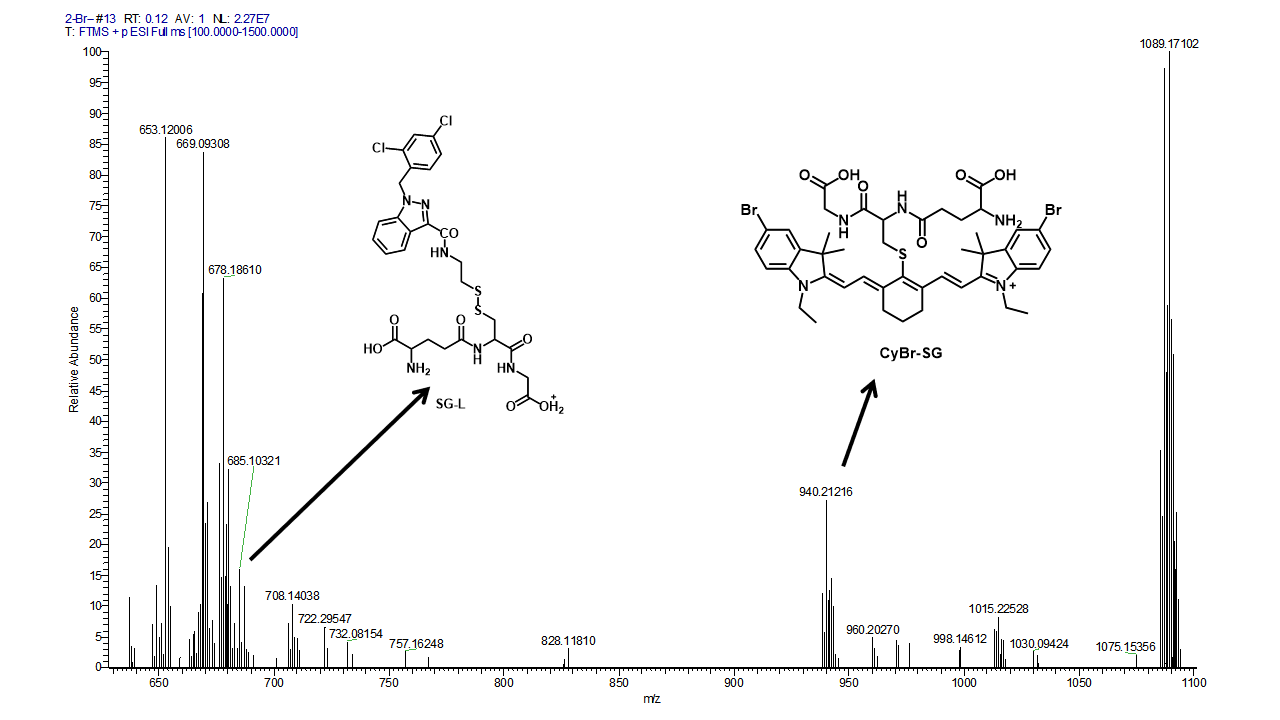


**Figure S11** ESI-HRMS of CyBr-SS-L in the GSH solution


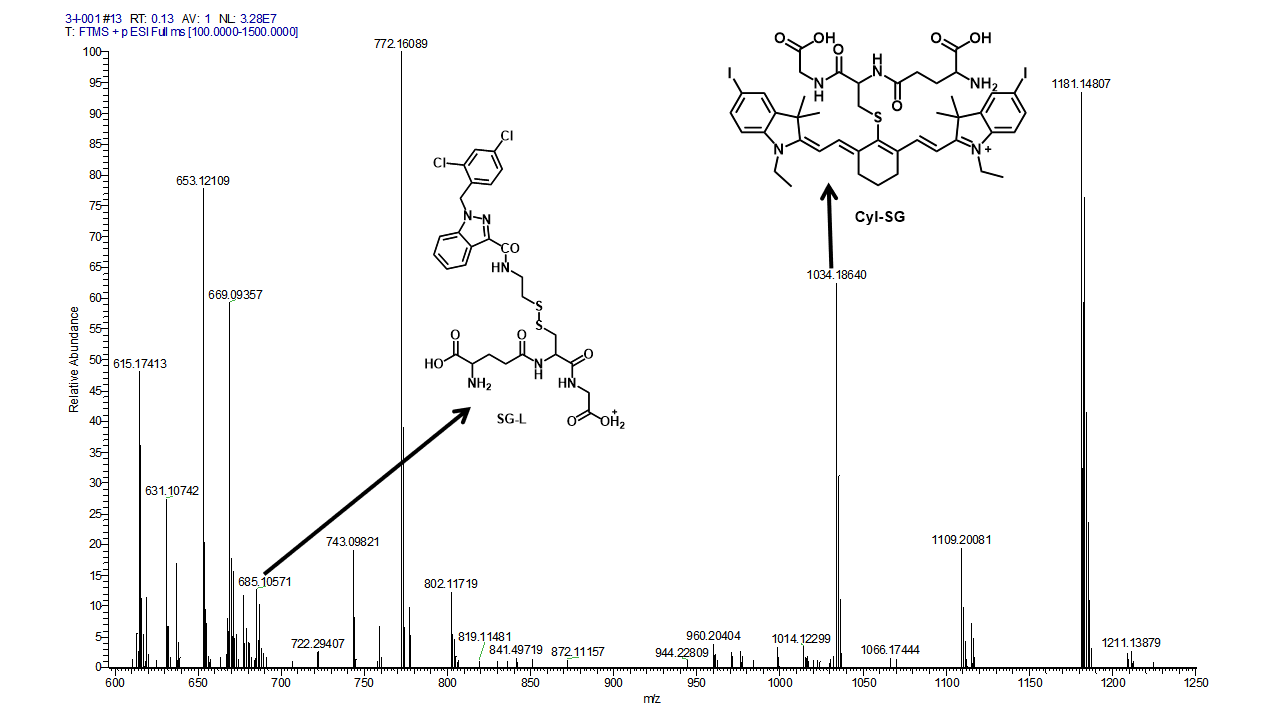


**Figure S12** ESI-HRMS of CyI-SS-L in the GSH solution


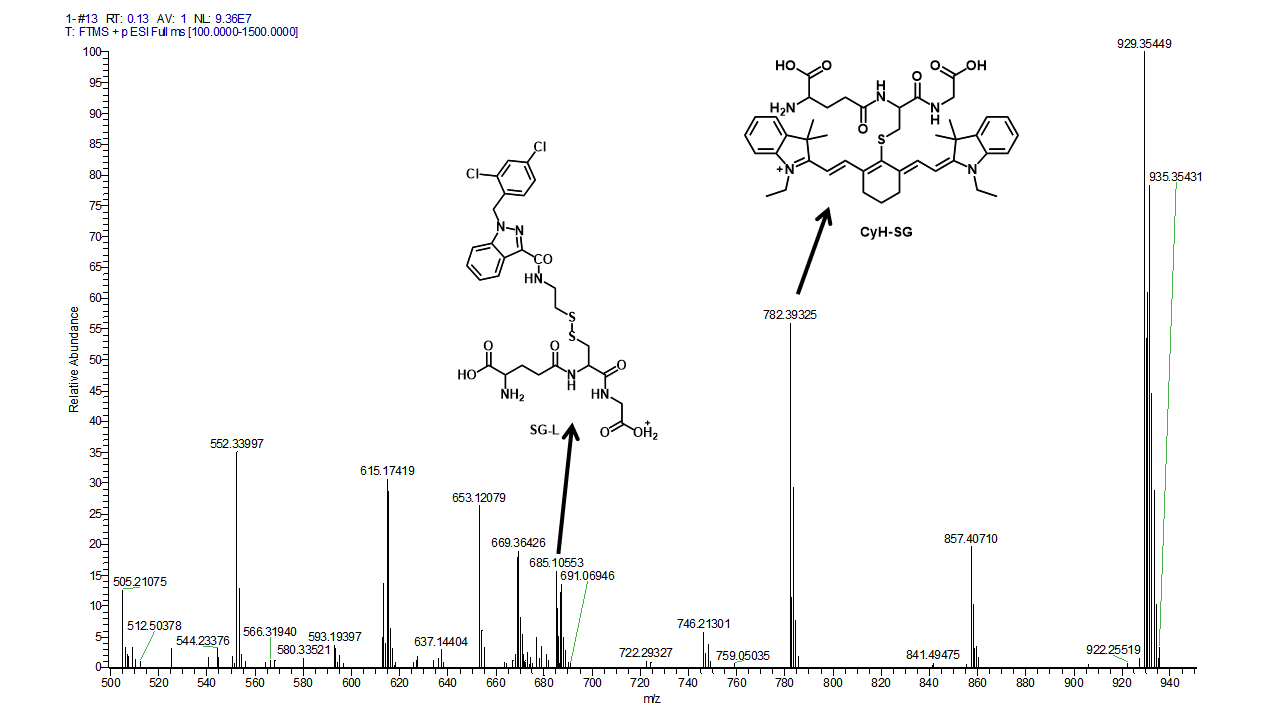


**Figure S13** ESI-HRMS of CyH-SS-L in the GSH solution


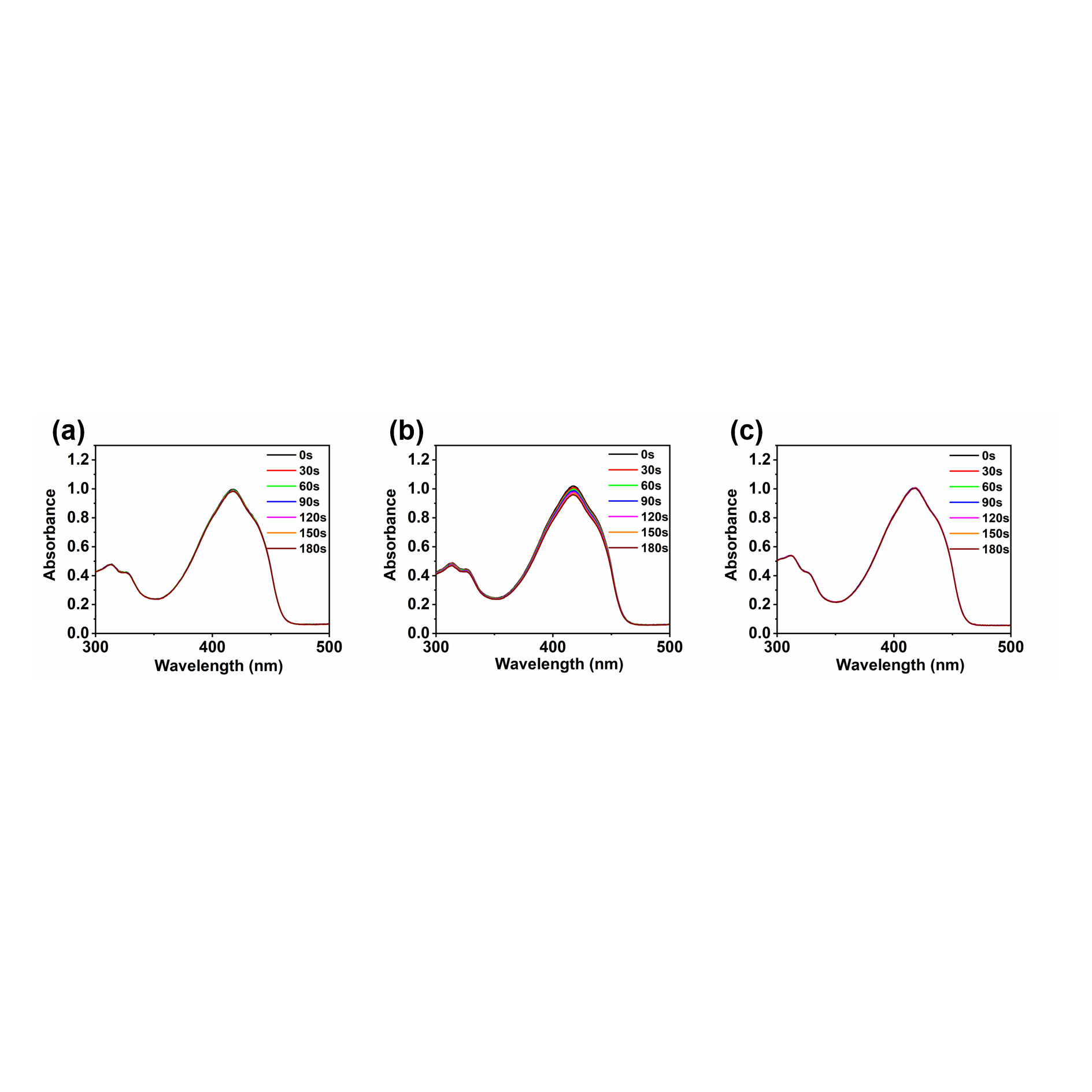


**Figure S14** Photodegradation curves of DPBF in the presence of **(a)** CyBr-SS-L (5 μM), **(b)** CyI-SS-L (5 μM), **(c)** CyH-SS-L (5 μM) under 808 nm light irradiation for different times (0-180 s)


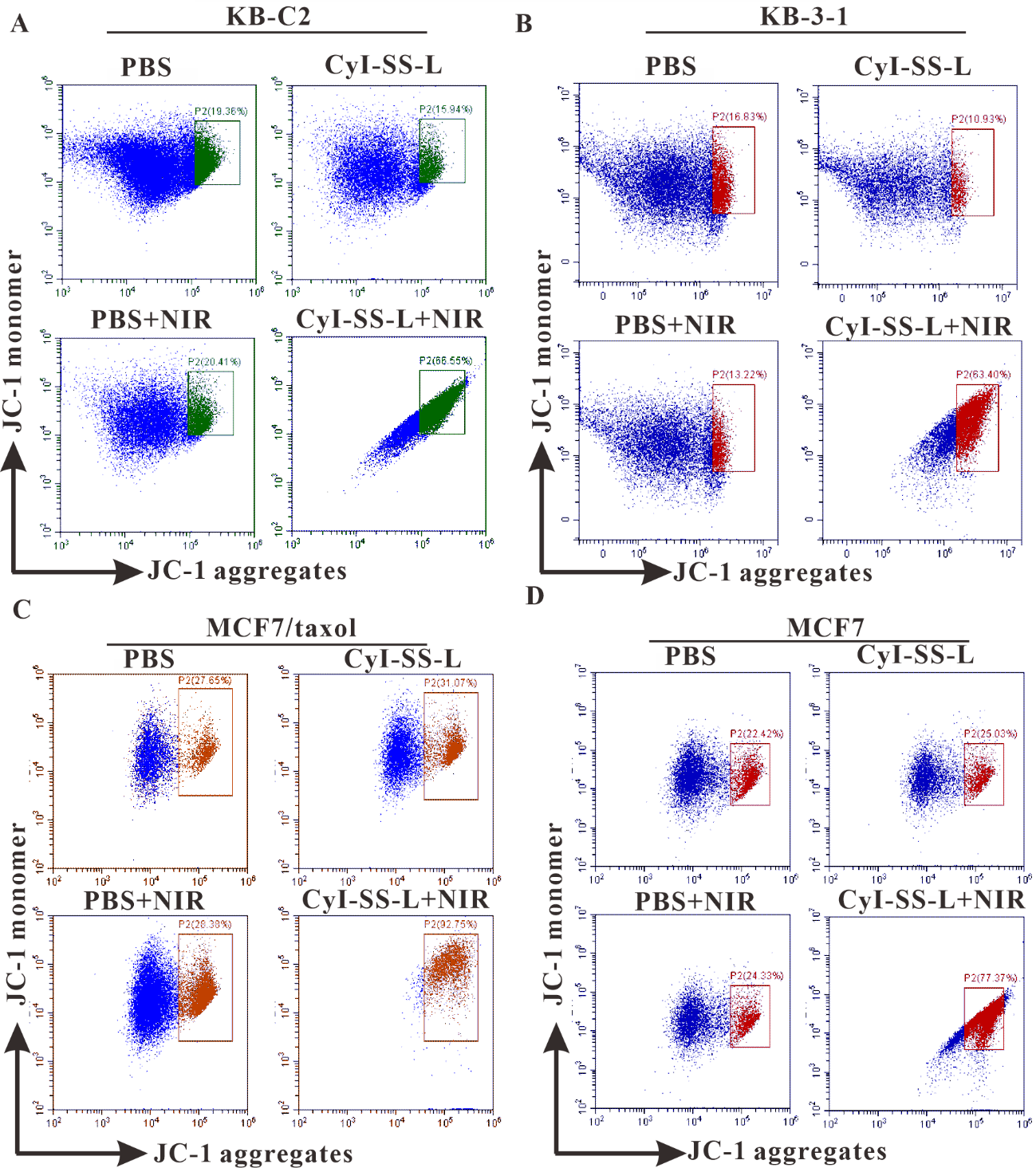


**Figure S15** The mitochondrial membrane potential changes in KB-C2 cells, KB-3-1 cells, MCF7/taxol cells, and MCF7 cells were detected using flow cytometry following various treatments

| **Table S1** Singlet oxygen yield (*Φ_△_*) of MB, ICG, CyH-SS-L, CyBr-SS-L, CyI-SS-L. | | |
| --- | --- | --- |
| **Photosensitizer** | **Slope** | ***Φ_△_*** |
| MB | -6.66×10^-2^ | 52.00% |
| ICG | -3.88×10^-3^ | 3.10% |
| CyH-SS-L | -2.73×10^-3^ | 2.14% |
| CyBr-SS-L | -5.30×10^-3^ | 4.02% |
| CyI-SS-L | -6.98×10^-3^ | 5.37% |
